# Radiolabeling efficiency of FENTA chelators and stability of their terbium-161, lutetium-177 and bismuth-213 complexes

**DOI:** 10.1186/s41181-026-00438-y

**Published:** 2026-04-07

**Authors:** Cédric Bonneux, Sunay Rodríguez Pérez, Stephan Heinitz, Veronique Bogaerts, Michiel Van de Voorde, Thomas Cardinaels, Maarten Ooms, Wim Dehaen, Tomas Opsomer

**Affiliations:** 1 Institute for Nuclear Medical Applications, Belgian Nuclear Research Centre (SCK CEN), Mol, Belgium; 2 Department of Chemistry, Sustainable Chemistry for Metals and Molecules, KU Leuven, Leuven, Belgium

**Keywords:** Terbium-161, Lutetium-177, Bismuth-213, Radiolabeling, Bifunctional chelator, H_4_FENTA, Chelators

## Abstract

**Background:**

Phenanthroline derivatives are well-known chelators in coordination chemistry, but their potential in the rapidly evolving field of targeted radionuclide therapy (TRT) has not yet been explored. In TRT, DOTA remains the gold standard chelator for several clinically relevant radionuclides such as terbium-161, lutetium-177 and bismuth-213. However, its requirement for elevated labeling temperatures is a drawback, particularly for heat-sensitive targeting vectors. Although several alternative chelators have been reported in recent years, there remains an interest in new systems with suitable complexation properties. In this work, we evaluate whether phenanthroline-based ligands can serve as useful chelators in TRT, using the octadentate chelator H_4_FENTA and a newly developed bifunctional analog, BF-FENTA. Their ability to complex [^161^Tb]Tb^3+^, [^177^Lu]Lu^3+^, and [^213^Bi]Bi^3+^, as well as their kinetic inertness, was assessed and compared to the benchmark chelators DOTA and CHX-A”-DTPA.

**Results:**

BF-FENTA was prepared via mono-substitution of 2,9-bis(chloromethyl)-1,10-phenanthroline with di-*tert*-butyl iminodiacetate, followed by a second substitution with the bifunctional arm. Both H_4_FENTA and BF-FENTA efficiently incorporated [^161^Tb]Tb^3+^ under mild conditions within 15 min at an apparent molar activity (AMA) of 150 MBq/nmol. Stability studies showed that both chelators formed an unstable complex with [^161^Tb]Tb^3+^, while the [^177^Lu]Lu^3+^ chelates showed similar stability compared to DOTA after 7 days in human serum. However, a DTPA challenge indicated a reduced kinetic inertness for both FENTA chelators compared to DOTA. For [^213^Bi]Bi^3+^, rapid incorporation was observed with the phenanthroline chelators, with H_4_FENTA achieving high radiochemical conversions (> 90%) at high AMAs of up to ~ 200 MBq/nmol after 5 min. Additionally, H_4_FENTA displayed a high selectivity for [^213^Bi]Bi^3+^ in the presence of competing metal ions. BF-FENTA showed slightly less favorable chelation properties with [^213^Bi]Bi^3+^ compared to H_4_FENTA. Nonetheless, the [^213^Bi]Bi^3+^ complexes remained intact in both buffer (NH_4_OAc, pH 6.0) and human serum after 90 min.

**Conclusion:**

Both H_4_FENTA and BF-FENTA rapidly incorporated terbium-161, lutetium-177, and bismuth-213. While the kinetic inertness of their terbium-161 and lutetium-177 complexes was inadequate, H_4_FENTA exhibited favorable kinetic inertness with bismuth-213 over the 90 min timeframe, identifying it as a promising chelator. In contrast, further structural refinement is needed for its bifunctional analog.

**Supplementary Information:**

The online version contains supplementary material available at 10.1186/s41181-026-00438-y.

## Introduction

Targeted radionuclide therapy (TRT) delivers therapeutic radionuclides in the form of radiopharmaceuticals directly to cancer cells, enabling highly localized radiation (Pouget et al. 2011). Commonly, a radiometal-based radiopharmaceutical consists of three main components: (1) a *β*^−^-, *α*-, or Auger emitting radionuclide, (2) a vector molecule (*e.g.* peptide, small molecule, antibody) that directs the radiopharmaceutical to its specific target, and (3) a bifunctional chelator that both coordinates the radiometal and links it to the targeting vector (Kostelnik and Orvig 2019).

Among the radionuclides, lutetium-177 (t_1/2_ = 6.65 d) (Pommé et al. 2011) has become the benchmark *β*^*−*^-emitter for TRT because of its favorable decay properties, as well as its optimized clinical production and multiple successes (Kostelnik and Orvig 2019; Tran et al. 2025; Lepareur 2025; Institute and Approval 2018; Institute 2022). A promising alternative is terbium-161 (t_1/2_ = 6.96 d) (Lisowska et al. 2026), which has similar coordination chemistry and nuclear decay properties, but has the benefit of emitting a significant amount of internal conversion and Auger-electrons. It is therefore hypothesized that terbium-161 has a higher therapeutic efficacy compared to lutetium-177 (Tran et al. 2025; Laere et al. 2024; Alcocer-Ávila et al. 2020; Buteau et al. 2025).

*α*-Emitters, such as bismuth-213 (t_1/2_ = 45.6 min) (Takács and Kossert 2021) and actinium-225 (t_1/2_ = 9.92 d) (Pommé et al. 2012), have gained a significant amount of attention because of their ability to induce more effective damage to tumor cells compared to *β*^*−*^*-*emitters, with limited damage to surrounding healthy cells (Pouget et al. 2011). A key advantage of bismuth-213 is that it can be eluted multiple times per day from an actinium-225/bismuth-213 generator, enabling a reliable local supply for several weeks. This, combined with several encouraging preclinical and clinical results, has positioned bismuth-213 as a promising radionuclide for targeted alpha therapy (TAT) (Franchi et al. 2022; Ahenkorah et al. 2021; Nawar et al. 2025).

**Fig. 1:**
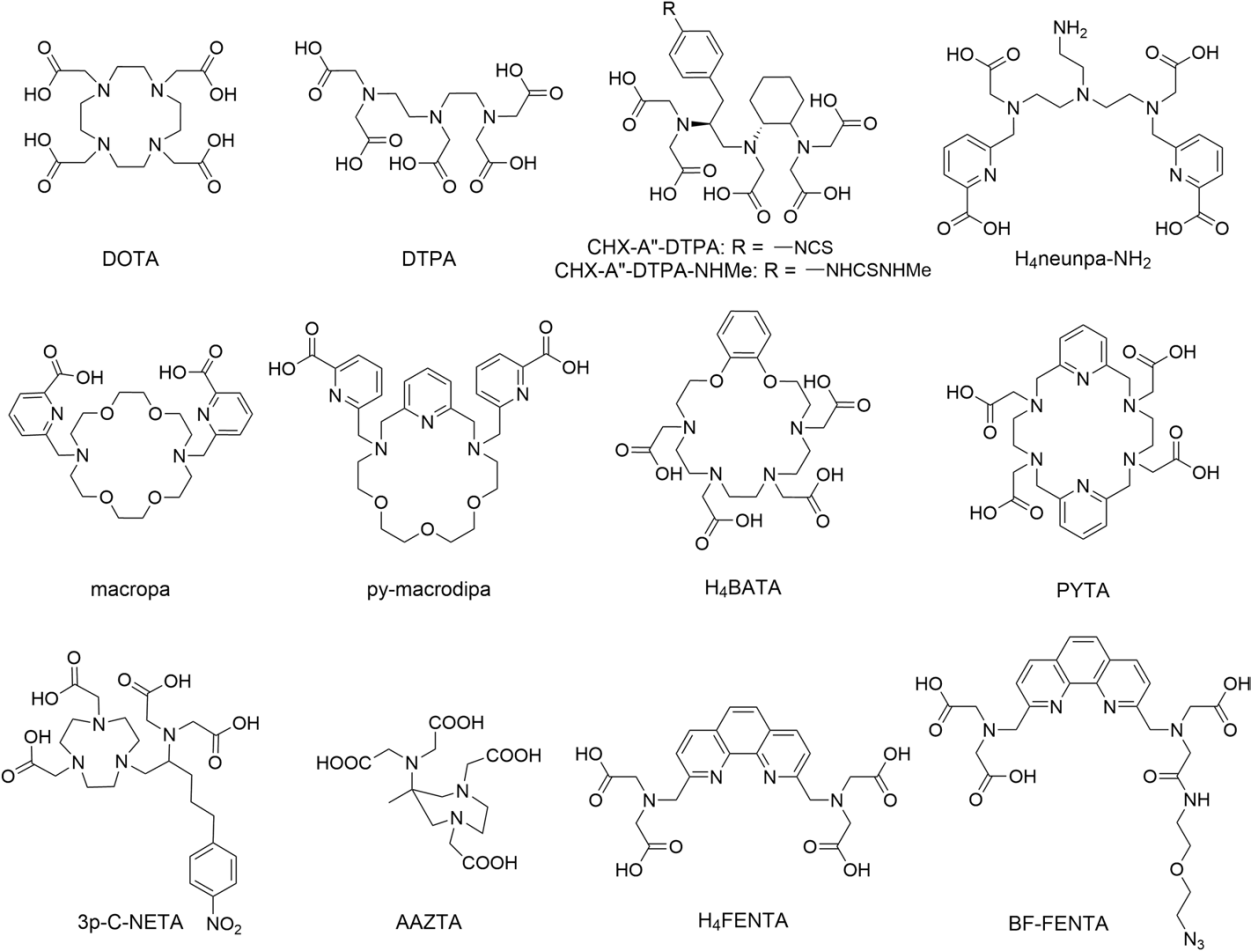
Chelators discussed in this work

Currently, the macrocyclic H_4_DOTA (1,4,7,10-tetraazacyclododecane-1,4,7,10-tetraacetic acid; Fig. 1) is considered the “gold standard” chelator for most therapeutic radiometals, including those mentioned above, because of the high stability of its complexes (Laere et al. 2024; Batool et al. 2025). However, DOTA typically requires elevated temperatures for labeling (Kostelnik and Orvig 2019; Batool et al. 2025; Cassells et al. 2021). This is undesirable in case of heat-sensitive targeting vectors, such as monoclonal antibodies. In contrast, acyclic chelators like DTPA (diethylenetriaminepentaacetic acid) generally offer more favorable labeling kinetics under mild conditions, but often lack the stability and kinetic inertness needed for TRT as they do not benefit from the macrocyclic effect. To compensate for this, a preorganized structure can be introduced along the backbone to enhance stability. For example, CHX-A”-DTPA complexes show improved stability compared to DTPA (Montavon et al. 2012; Stimmel and Kull 1998). However, they still demonstrate moderate kinetic inertness relative to the DOTA complexes. Consequently, in recent years, various chelators have been explored with the goal of improving labeling kinetics while preserving high complex stability. Among them, open chain chelators such as H_4_neunpa-NH_2_ have been studied (Wharton et al. 2022, 2021). In addition, hybrid chelators that combine the preorganization of macrocycles with the flexibility of acyclic systems such as bispidine-based ligands (Kopp et al. 2023; Cieslik et al. 2022, 2024; Bruchertseifer et al. 2020; Bleher et al. 2025), AAZTA (6-amino-6-methylperhydro-1,4-diazepinetetraacetic acid) (Pfister et al. 2015; Sinnes et al. 2019; Horváth et al. 2022) and 3p-C-NETA (2,2'-((1-(4,7-bis(carboxymethyl)-1,4,7-triazonan-1-yl)-5-(4-nitrophenyl)pentan-2-yl)azanediyl)diacetic acid) (Ahenkorah et al. 2022; Kang et al. 2013; Chong et al. 2011) have been investigated. Another class of chelators is based on aza-18-crown-6 ether derivatives. The enlarged macrocycles have been shown to combine rapid labeling with high stability. A few examples are macropa (Roca-Sabio et al. 2009; Fiszbein et al. 2021; Thiele et al. 2017; Simms et al. 2024), py-macrodipa (Hu et al. 2022, 2021), H_4_BATA (benzodioxatetraaza-18-crown-6 tetraacetic acid) (Matazova et al. 2022, 2019), and PYTA (3,6,10,13-tetraaza-1,8(2,6)-dipyridinacyclotetradecaphane-3,6,10,13-tetraacetic acid) (Simms et al. 2024; Faltejsek et al. 2025). Several of these systems have already shown highly promising results in combination with various radionuclides, reflecting the ongoing progress in chelator development.

An interesting class of ligands are 1,10-phenanthrolines, because of their good chelation properties with a wide range of metal ions. Phenanthroline rigid pre-organized structure entropically favors complex formation (Bencini and Lippolis 2010; Queffélec et al. 2024). The addition of chelating substituents at positions 2 and 9 increases the denticity, enabling the formation of stable complexes with metals such as the lanthanides, which typically require coordination numbers of 8 or higher. One such modified phenanthroline derivative is H_4_FENTA (2,2ʹ,2″,2‴-(((1,10-phenanthroline-2,9-diyl)bis(methylene))bis(azanetriyl))tetraacetic acid; Fig. 1). H_4_FENTA was first synthesized by Mukkala et al*.* to evaluate the luminescence properties of its complexes with Tb(III) and Eu(III) (Mukkala et al. 1992). Later, these complexes were also studied by Lin et al*.* and Zhang et al*.*, with the latter connecting H_4_FENTA to silica nanoparticles (Lin et al. 2002a, 2002b; Zhang et al. 2007). More recently, Váradi et al*.* investigated the coordination properties of H_4_FENTA with Gd(III) (Váradi et al. 2022). Their results showed a similar stability of [Gd(FENTA)]^−^ at physiological pH compared to [Gd(DOTA)]^−^ and [Gd(DTPA)]^2−^. These findings highlight the potential of H_4_FENTA as a chelator for radiolanthanides. Interestingly, while phenanthroline-based ligands have seen significant use in coordination chemistry, they have yet to be explored in TRT.

In this context, we evaluated the suitability of the acyclic chelator H_4_FENTA and its first bifunctional analog, BF-FENTA, for the stable chelation of therapeutic radiometals. Their radiolabeling efficiency and kinetic inertness are assessed with [^161^Tb]Tb^3+^, [^177^Lu]Lu^3+^, and [^213^Bi]Bi^3+^, and the results are compared to the benchmark chelators DOTA and CHX-A”-DTPA. These studies provide insight into the potential of phenanthroline-derived acyclic chelators in TRT.

## Results

### Synthesis of H_4_FENTA and BF-FENTA

**Scheme 1:**
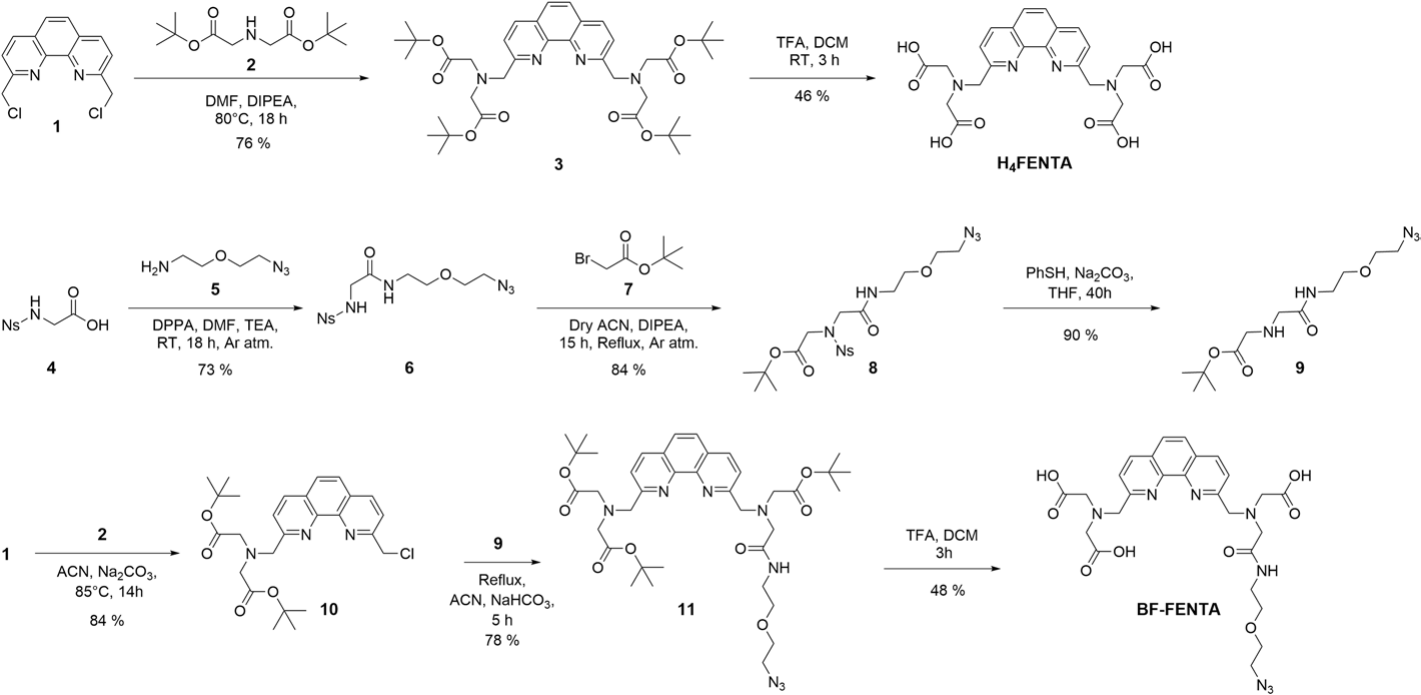
Synthetic routes for H_4_FENTA and BF-FENTA

H_4_FENTA was synthesized using a modified reported procedure (Scheme 1) (Mukkala et al. 1992). (tBu)_4_FENTA **3** was obtained in a relatively good yield (76%) via the S_N_2 reaction of 2,9-bis(chloromethyl)-1,10-phenanthroline **1** instead of the corresponding dibromide (Weijnen and Engbersen 1993a), with di-*tert*-butyl iminodiacetate **2**.

To synthesize BF-FENTA containing an azide group for further conjugation, a stepwise approach was used. The mono-substituted phenanthroline **10** was prepared by reacting two equivalents of 2,9-bis(chloromethyl)-1,10-phenanthroline **1** with one equivalent of di-*tert*-butyl iminodiacetate **2**, affording **10** in 84% yield with 92% recovery of the unreacted dichloride **1**. BF-FENTA was then obtained after reacting compound **10** with amine **9** and subsequent acidic removal of the tert-butyl groups, in 37% yield over two steps.

### Radiolabeling and stability experiments with terbium-161 and lutetium-177

**Fig. 2:**
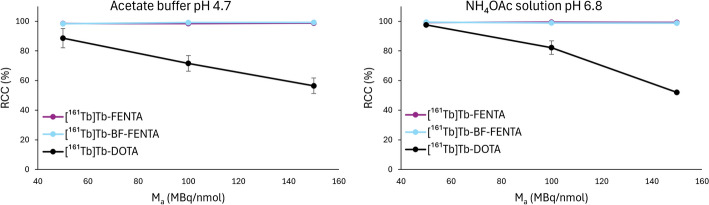
Molar activity escalation studies of H_4_FENTA, BF-FENTA, and DOTA with [^161^Tb]Tb^3+^ at left: pH 4.7 (0.1 M NaOAc buffer); right: pH 6.8 (0.5 M NH_4_OAc solution). All reactions were carried out in a total volume of 60 µL in the presence of ascorbic acid (AA; 8.3 mM) with 10 MBq [^161^Tb]Tb^3+^ at 40 °C. The RCCs were evaluated after 15 min by iTLC analysis and quantified using a gamma counter (cut and count method; n = 3)

An initial molar activity escalation study was conducted to evaluate the suitability of H_4_FENTA and BF-FENTA for chelating [^161^Tb]Tb^3+^ (Fig. 2). Radiolabeling was performed using 10 MBq [^161^Tb]Tb^3+^ in two buffer systems (0.1 M NaOAc buffer, pH 4.7; and 0.5 M NH_4_OAc solution, pH 6.8) under mild conditions (40 °C, 15 min), which are compatible with thermolabile vector molecules. Both H_4_FENTA and BF-FENTA exhibited quantitative radiochemical conversions (RCCs > 99%) under all evaluated conditions with chelator concentrations ranging from 3.33 µM to 1.11 µM. These concentrations correspond to an apparent molar activity (AMA) range of 50–150 MBq/nmol and ligand-to-radiometal ratios (L/M) between 14 and 5, respectively. In contrast, DOTA showed RCCs decreasing from 87 ± 7% to 57 ± 5% in NaOAc buffer (pH 4.7), and from 98 ± 1% to 52 ± 0.3% in the NH_4_OAc solution (pH 6.8), when increasing the AMA from 50 to 150 MBq/nmol, respectively.

**Fig. 3:**
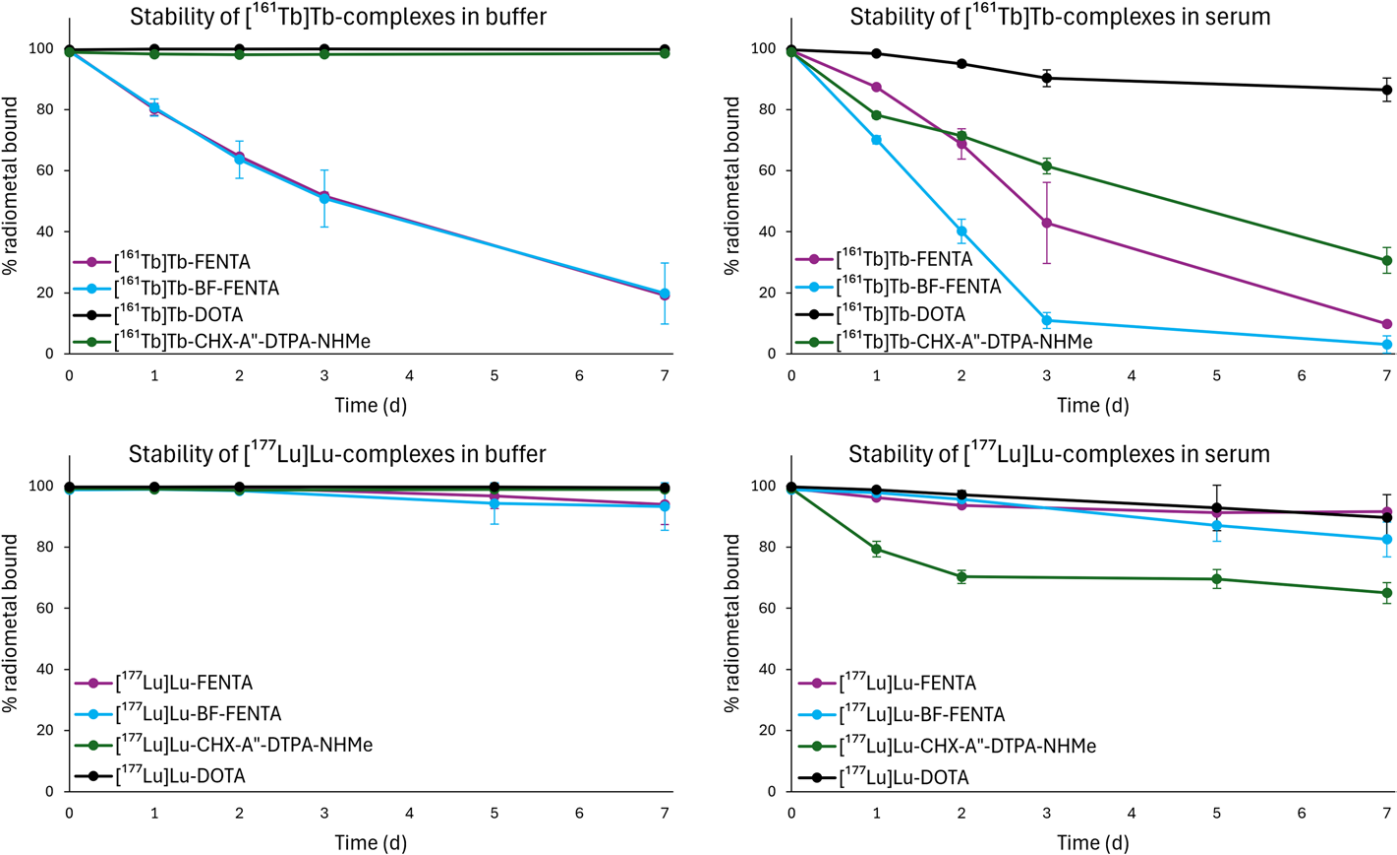
Stability of [^161^Tb]Tb^3+^ (top) and [^177^Lu]Lu^3+^ (bottom) complexes of H_4_FENTA, BF-FENTA, CHX-A”-DTPA-NHMe and DOTA in NaOAc buffer (0.25 M, pH 4.7) at RT (left) and in human serum at 37 °C (right). All reactions were carried out at an AMA of 50 MBq/nmol in the presence of AA (33 mM) at 40 °C for 15 min, except for the reactions with DOTA (85 °C, 15 min). After radiolabeling, the solution was diluted 3.33-fold with acetate buffer or 4-fold with serum. The percentage of radiometal bound was determined by iTLC analysis and quantified using a gamma counter (cut and count method; n = 3)

Next, we evaluated the kinetic inertness of the [^161^Tb]Tb^3+^ and [^177^Lu]Lu^3+^ complexes in radiolabeling buffer with and without ascorbic acid (AA), and in human serum (Figs. 3, S6–S9) (Hunt et al. 2025). The percentage of radiometal still bound to the chelator was evaluated by TLC analysis over a 7-day period, aligning with the physical half-lives of these radionuclides. Despite their efficient radiolabeling, both [^161^Tb]Tb-FENTA and [^161^Tb]Tb-BF-FENTA showed limited stability in buffer, with only 19 ± 1% and 20 ± 10% of the complexes remaining intact after 7 days, respectively (Fig. 3). In contrast, over 98% of [^161^Tb]Tb-DOTA and [^161^Tb]Tb-CHX-A”-DTPA-NHMe remained intact under identical conditions. Interestingly, no significant stability differences were observed between 0 and 10 mM AA for FENTA and BF-FENTA radiocomplexes over time (Figs. S6, S8).

A similar trend was observed in human serum. The FENTA-based chelators showed poor kinetic inertness, with just 10 ± 1% and 3 ± 3% of intact complex remaining for [^161^Tb]Tb-FENTA and [^161^Tb]Tb-BF-FENTA, respectively. Leaching was also observed for the open chain chelator CHX-A”-DTPA-NHMe, retaining 31 ± 4% of [^161^Tb]Tb^3+^ after 7 days. Unlike the other chelators, the DOTA complex was less affected by the serum proteins, with 86 ± 4% remaining intact.

In comparison, the corresponding [^177^Lu]Lu^3+^ complexes with the phenanthroline-based chelators showed higher stability. After 7 days in buffer, [^177^Lu]Lu-FENTA and [^177^Lu]Lu-BF-FENTA remained largely intact (93 ± 7% and 93 ± 8%, respectively), demonstrating stability comparable to the DOTA and CHX-A”-DTPA-NHMe complexes. Again, no difference in buffer stability was observed between 0 and 10 mM AA formulations (Figs. S7, S9).

In human serum, CHX-A”-DTPA-NHMe showed the lowest kinetic inertness of all chelators with [^177^Lu]Lu^3+^, retaining 65 ± 3% of radiometal after 7 days. In contrast, [^177^Lu]Lu-FENTA demonstrated high kinetic inertness under physiological conditions (92 ± 1%), matching DOTA (90 ± 7%). BF-FENTA, however, showed a slightly reduced serum stability (83 ± 6%).

To further evaluate the kinetic inertness, a DTPA challenge was performed by adding a 1000-fold excess of DTPA, with respect to the amount of chelator, to [^161^Tb]Tb^3+^ and [^177^Lu]Lu^3+^ complexes of H_4_FENTA, BF-FENTA and CHX-A”-DTPA-NHMe. Transchelation was evaluated by radio-HPLC analysis over a 7-day period (Figs. S10, S11). Radio-HPLC was used because the DTPA complexes could not be separated from the FENTA complexes by iTLC. DOTA complexes did not show any retention under the used HPLC conditions, and were therefore not included in this study. After 7 days, 57 ± 17% of [^177^Lu]Lu-H_4_FENTA and 26 ± 6% of [^177^Lu]Lu-BF-FENTA remained intact, whereas no intact CHX-A”-DTPA-NHMe complex was detected. For [^161^Tb]Tb^3+^, an even faster decrease of the percentage of intact complex was observed for all chelators.

### Radiolabeling and stability experiments with bismuth-213

Radiolabeling studies were conducted with 1.1–2.5 MBq [^213^Bi]Bi^3+^ at activity concentrations ranging from 18 to 27 MBq/mL, allowing multiple radiolabelings to be performed from a single eluate while maintaining reliable quantification of iTLC results. Considering the short half-life of this radionuclide, an initial association experiment was performed with and without AA to determine the required reaction time. Both FENTA and BF-FENTA demonstrated rapid complex formation, obtaining quantitative yields within 1 min in NaOAc buffer (1 M, pH 4.8) at an AMA of 2.2–2.3 MBq/nmol (Fig. S13). Quantitative RCCs were obtained in the presence and absence of 30 mM AA.

**Fig. 4:**
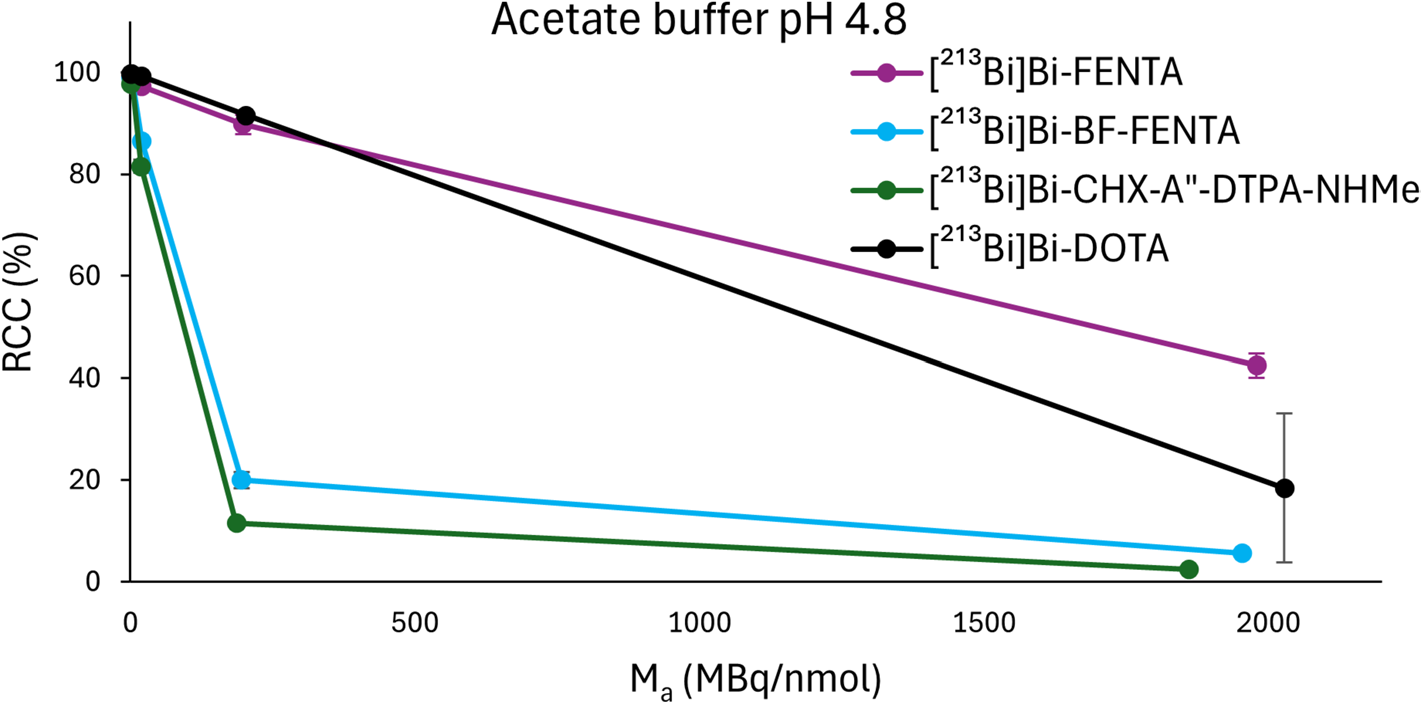
Molar activity escalation experiment of H_4_FENTA, BF-FENTA, DOTA, and CHX-A”-DTPA-NHMe with [^213^Bi]Bi^3+^ (1.1–1.2 MBq, 40 µL). All reactions were carried out in 1 M NaOAc buffer (pH 4.8, 20 µL) in the presence of AA (30 mM), at 40 °C for 5 min. For DOTA, labeling was performed at 90 °C for 30 min (n = 3). The RCCs were determined by iTLC analysis and quantified using a gamma counter (cut and count method; n = 3)

To further assess labeling efficiency with [^213^Bi]Bi^3+^, molar activity escalation studies were performed with 1.1 – 1.2 MBq [^213^Bi]Bi^3+^ in NaOAc buffer (1 M, pH 4.8). RCCs as a function of the AMA are depicted in Fig. 4 and RCCs as a function of the ligand concentration in Fig. S14. Quantitative RCCs were obtained for all chelators at an AMA of ~ 2 MBq/nmol, but differences became apparent at higher AMAs. Among the chelators evaluated, H_4_FENTA achieved similar results compared to DOTA, maintaining high RCCs (> 90%) up to an AMA of ~ 200 MBq/nmol. This corresponds to a L/M of ~ 762 and a chelator concentration of 10^–7^ M during radiolabeling. Notably, no high temperatures were required for H_4_FENTA to achieve these conversions, whereas DOTA required heating to 90 °C for 30 min. At ~ 2000 MBq/nmol (L/M of ~ 76), H_4_FENTA and DOTA showed limited incorporation of [^213^Bi]Bi^3+^, achieving RCCs of 42 ± 2% and 18 ± 15%, respectively. BF-FENTA demonstrated a lower radiolabeling efficiency with [^213^Bi]Bi^3+^ compared to H_4_FENTA, incorporating 86 ± 1% at ~ 20 MBq/nmol and only 20 ± 2% at ~ 200 MBq/nmol, similar to CHX-A”-DTPA-NHMe.

**Fig. 5:**
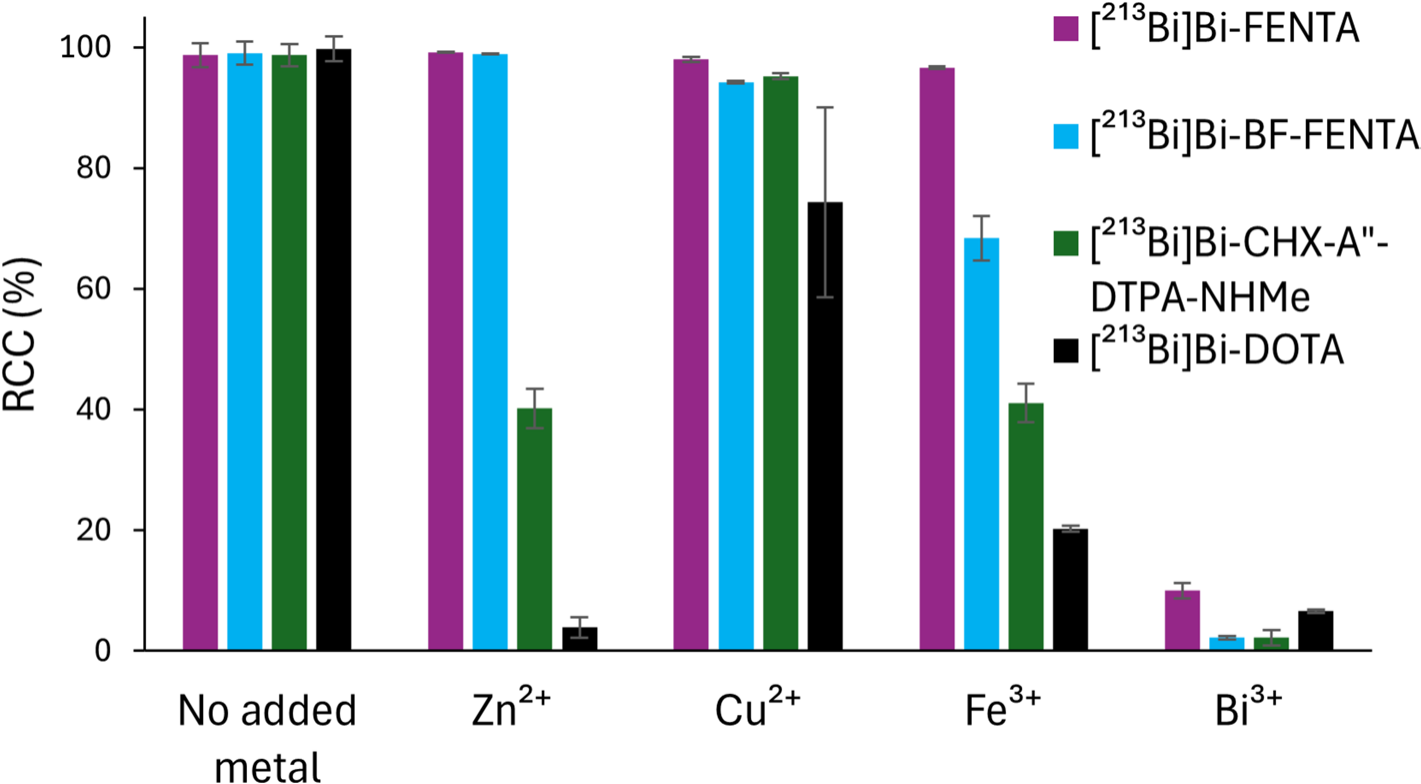
Metal challenge study where Zn^2+^, Cu^2+^, Fe^3+^, and Bi^3+^ (10^–4^ M) were added to H_4_FENTA, BF-FENTA, CHX-A”-DTPA-NHMe, and DOTA (10^–5^ M) prior to the addition of [^213^Bi]Bi^3+^ (1.2–1.4 MBq, 40 µL). All reactions were carried out in 1 M NaOAc buffer (pH 4.8, 20 µL) in the presence of AA (30 mM), at 40 °C for 5 min. For DOTA, labeling was performed at 90 °C for 30 min. The RCCs were determined by iTLC analysis and quantified using a gamma counter (cut and count method; n = 3)

To evaluate the chelation selectivity for Bi^3+^, a metal challenge experiment was performed by adding a 10-fold excess of competing metal ions (Zn^2+^, Cu^2+^, Fe^3+^) relative to the chelator (10^–5^ M) before [^213^Bi]Bi^3+^ (1.3–1.4 MBq; Fig. 5). Bi^3+^ was included as a control experiment. H_4_FENTA demonstrated high selectivity for [^213^Bi]Bi^3+^, with RCCs exceeding 96% in the presence of either Zn^2+^, Cu^2+^ or Fe^3+^_._ BF-FENTA showed a similar selectivity. However, only a moderate RCC was obtained in the presence of Fe^3+^ (68 ± 4%). Nonetheless, both chelators outperformed both DOTA and CHX-A”-DTPA-NHMe in this study.

**Fig. 6:**
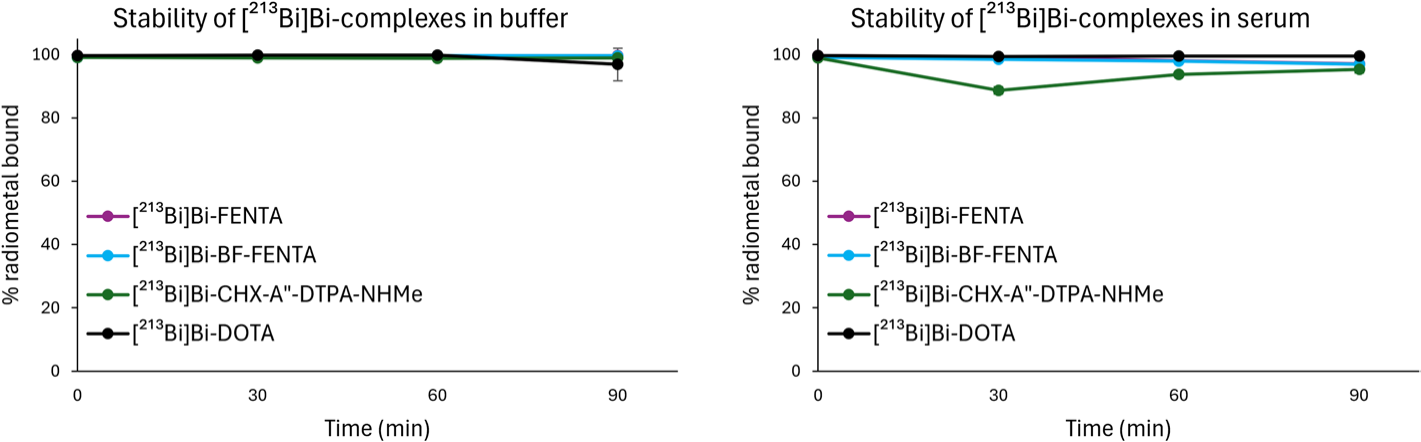
Stability of [^213^Bi]Bi^3+^-complexes in NaOAc buffer (1 M, pH 4.8) at RT (left) and in human serum at 37 °C (right). All reactions were carried out at an AMA of 2.4–2.7 MBq/nmol in the presence of AA (30 mM), at 40 °C for 5 min, except for the reactions with DOTA (90 °C, 30 min). After radiolabeling, the solution was diluted twofold with serum. iTLC strips were analyzed by autoradiography and the percentage of radiometal bound was quantified using a gamma counter (cut and count method; n = 3)

Next, the stability of the [^213^Bi]Bi^3+^ complexes was monitored in NaOAc buffer and human serum over a 90-min period, corresponding to two half-lives (Fig. 6). Notably, all chelators demonstrated to remain intact over this period.

## Discussion

### Synthesis

In this work, we described the synthesis of H_4_FENTA from 2,9-bis(chloromethyl)-1,10-phenanthroline **1** instead of the corresponding dibromide (Mukkala et al. 1992). Literature reports indicate that (tBu)_4_FENTA **3** obtained from the dibromide can be isolated as a pure product by simple filtration without chromatographic purification (Weijnen and Engbersen 1993a). However, we observed an impure product. (tBu)_4_FENTA **3** was therefore purified using a neutral alumina cartridge, affording the product in a relatively good yield when starting from the dichloride **1** (76%), while a slightly lower yield was obtained when using the dibromide (66%).

The bifunctional derivative BF-FENTA was synthesized via mono-substitution of 2,9-bis(chloromethyl)-1,10-phenanthroline with di-*tert*-butyl iminodiacetate **2**, resulting in phenanthroline **10** with one chloromethyl. Alternatively, the corresponding bromomethyl analog was also prepared. However, this compound was obtained in low yield (< 25%) due to its poor stability, as confirmed by 2D TLC analysis. For the subsequent synthesis of tri-*tert*-butyl-BF-FENTA **11**, heating should not be performed for longer than 5 h, as prolonged heating at reflux led to the formation of an undesired 2,6-piperazinedione. Notably, the azide group in BF-FENTA was introduced as a reactive handle. It enables conjugation to a vector molecule via a strain-promoted azide–alkyne cycloaddition reaction and can also be reduced to an amine through Staudinger reduction.

### Radiolabeling with terbium-161 and lutetium-177

In the molar activity escalation study, both H_4_FENTA and BF-FENTA achieved quantitative RCCs with [^161^Tb]Tb^3+^ at a low L/M of 5 under mild conditions. In contrast, DOTA did not reach quantitative conversion, as elevated temperatures are typically required for this chelator (Cassells et al. 2021). However, near-quantitative conversion (98%) was obtained for DOTA at pH 6.8 and 50 MBq/nmol, highlighting the impact of both pH and molar activity on the radiolabeling efficiency. At 50–100 MBq/nmol, higher RCCs were observed in the NH_4_OAc solution (pH 6.8), whereas at 150 MBq/nmol, a similar RCC was observed in NaOAc buffer (pH 4.7). This may reflect partial hydrolysis of Tb^3+^ at low DOTA concentrations and increased pH (Brown and Ekberg 2016). Since radiolabeling of DOTA with [^161^Tb]Tb^3+^ is generally not performed above pH 6.0 (Lehenberger et al. 2011; McNeil et al. 2022), NaOAc buffer was used in further studies. Overall, the results demonstrated that the open chain chelators H_4_FENTA and BF-FENTA could efficiently incorporate [^161^Tb]Tb^3+^ under mild conditions at high AMAs.

Despite favorable labeling properties, [^161^Tb]Tb^3+^ complexes with H_4_FENTA and BF-FENTA were unstable in both buffer and serum, whereas [^177^Lu]Lu^3+^ complexes showed greater kinetic inertness. Because of the higher charge density, Lu^3+^ often forms more stable complexes than Tb^3+^, which may partly account for the observed differences in kinetic intertness (Nizou et al. 2020; Cacheris et al. 1987). Additionally, the observed difference in kinetic inertness between the [^161^Tb]Tb^3+^ and [^177^Lu]Lu^3+^ complexes might be attributed to a different coordination geometry. Supporting this hypothesis, radio-HPLC analysis revealed a significant difference in retention times between [^161^Tb]Tb-FENTA and [^177^Lu]Lu-FENTA (3.0 min and 5.1 min, respectively; Fig. S4). A large difference in retention, however, was not observed for the BF-FENTA radiocomplexes (Fig. S5).

For the radiolabelings with [^161^Tb]Tb^3+^ and [^177^Lu]Lu^3+^, AA was added as a commonly used radiolytic quencher in clinical formulation protocols, reaching final concentrations of 5.5–10 mM after dilution for stability testing. Since these concentrations are not expected to fully prevent radiolysis, buffer stability was evaluated in the absence of AA, which did not negatively affect the results for both radionuclides (Figs. S6–S9). This could indicate that the quencher formulation requires further optimization, particularly for clinical translation, or that non-radiolytic degradation pathways also play a role. Additional studies are needed to clarify these observations.

Notably, [^177^Lu]Lu-BF-FENTA was less stable than [^177^Lu]Lu-FENTA in serum. This reduced kinetic inertness has also been observed for DOTA-monoamide, compared to DOTA with four carboxylates, demonstrating that the amide group is a weaker donor (Weineisen et al. 2014; Tircsó et al. 2020; Tei et al. 2015). The DTPA challenge further confirmed this trend as H_4_FENTA outperformed BF-FENTA in this experiment. However, [^177^Lu]Lu-FENTA itself still underwent significant transchelation in the presence of DTPA, retaining only 57 ± 17% radiometal after 7 days. By comparison, [^177^Lu]Lu-DOTA-rituximab remained 97% intact under similar conditions in the presence of a 10^5^-fold excess of Ca-DTPA (Forrer et al. 2009). These results show that, although H_4_FENTA and BF-FENTA can form relatively stable complexes with [^177^Lu]Lu^3+^ in buffer and serum, they lack the kinetic inertness of DOTA. The results also imply that the preorganization of the FENTA-based chelators does not compensate for the absence of the macrocyclic effect. Therefore, it was concluded that FENTA-based complexes with Tb^3+^ and Lu^3+^ are not suitable for in vivo applications. Structural changes to H_4_FENTA, increasing the denticity, would be needed to address this limitation. Literature examples demonstrate that 18-membered pyridine-containing macrocycles have fast labeling kinetics, while maintaining a high complex stability (Simms et al. 2024; Hu et al. 2022, 2021; Faltejsek et al. 2025).

### Radiolabeling with bismuth-213

The short half-life of bismuth-213 requires rapid chelation of this radionuclide. Both FENTA-chelators met this requirement, with both being able to incorporate [^213^Bi]Bi^3+^ quantitatively within one minute. Next, during a molar activity escalation experiment, H_4_FENTA achieved a high RCC (> 90%) with [^213^Bi]Bi^3+^ at a ligand concentration of 0.1 µM and an AMA of 198 MBq/nmol (L/M = 770). While similar results have been reported for other chelators in literature at this ligand concentration (Fiszbein et al. 2021; Šimeček et al. 2018; Ingham et al. 2022; Wharton et al. 2023; Randhawa et al. 2024; Hierlmeier et al. 2025), these conversions were not achieved at a comparable AMA, with the exception of H_4_neunpa-NH_2_ (Wharton et al. 2022) (650 MBq/nmol). However, comparisons with literature data should be made with caution, as radiolabeling solutions with higher activity concentrations enable higher AMAs due to reduced interference from metal impurities. This also highlights the importance of including reference chelators in the experimental setup. DOTA exhibited RCCs comparable to H_4_FENTA. However, quantitative labeling required heating to 90 °C for 30 min. In contrast, BF-FENTA performed similarly to CHX-A”-DTPA-NHMe but inferior to H_4_FENTA, achieving a RCC of 20% at an AMA of 195 MBq/nmol, likely due to the weaker coordination of the amide. To investigate the cause of differing chelator performance in the molar activity escalation study, we evaluated the selectivity for Bi^3+^ over transition metal impurities (Fig. 5). These ions (Zn^2+^, Cu^2+^, Fe^3+^) are commonly present in both the environment and the body. In this study, both FENTA-chelators significantly outperformed the reference chelators and showed high selectivity for [^213^Bi]Bi^3+^. While BF-FENTA showed a moderate decrease in RCC (68 ± 4%) in the presence of Fe^3+^ compared to H_4_FENTA, similar RCCs were observed for both phenanthroline chelators with Cu^2+^ and Zn^2+^. As expected, the addition of Bi^3+^ caused a decrease in RCC below 10% for all chelators. However, these data do not explain the large difference in RCCs achieved with FENTA chelators at higher molar activities, implying that metal impurities were not the cause. Given DOTA’s high susceptibility to transition metal impurities, the high RCCs it achieved in the molar activity escalation study indicate that only trace levels of such impurities were present in our radiolabeling solutions. To our knowledge, competition experiments evaluating chelator selectivity for Bi^3+^ over metal impurities using [^213^Bi]Bi^3+^ have not yet been reported, with the exception of Hierlmeier et al., who reported the selective chelation of [^213^Bi]Bi^3+^ from ^225^Ac solutions by DOTI-TVA, a cyclen-based chelator with four (imidazol-2-yl)methyl substituents (Hierlmeier et al. 2025).

In addition to the selectivity of the phenanthroline chelators for Bi^3+^, their [^213^Bi]Bi^3+^ complexes also remained stable over 90 min in both buffer and human serum. Although AA was added to the radiolabelings, its absence did not reduce the RCCs or stability, indicating that radiolysis was not an issue at the activity concentrations used over the 90 min timeframe (Fig. S15). The results highlight H_4_FENTA as a promising chelator for [^213^Bi]Bi^3+^. However, a different bifunctional analog might be preferred for in vivo applications, because BF-FENTA underperformed in terms of radiolabeling efficiency. Similar to the DOTA-GA chelator, a suggestion would be to replace one acetate arm by an *α-*substituted glutarate (Eisenwiener et al. 2000). Another suggestion would be to functionalize the phenanthroline core. However, this could be synthetically more challenging. For instance, the synthesis of 2,9-di(bromomethyl)-5-nitro-1,10-phenanthroline from neocuproine has an overall yield of 1% (Kazumi et al. 1991). It is also important to note that conjugation to biomolecules may affect the chelator’s local environment, potentially reducing radiolabeling efficiency and in vivo stability. Combined with possible effects on the pharmacokinetic properties of the carrier, these factors may pose additional challenges in the application of this chelator.

## Conclusion

In this study, H_4_FENTA and its first bifunctional analog BF-FENTA were successfully synthesized, with BF-FENTA being prepared through two consecutive substitutions that allowed the introduction of a versatile azide group.

The phenanthroline-based chelators were then evaluated for their radiolabeling efficiency and in vitro stability using [^161^Tb]Tb^3+^, [^177^Lu]Lu^3+^, and [^213^Bi]Bi^3+^. Both chelators exhibited rapid complexation with [^161^Tb]Tb^3+^ at high AMAs. However, the resulting complexes displayed very poor kinetic inertness in buffer and serum, hindering their suitability for TRT applications with this radionuclide.

In contrast, [^177^Lu]Lu^3+^ complexes of both phenanthroline chelators showed better kinetic inertness in buffer, comparable to CHX-A”-DTPA-NHMe and DOTA. Both H_4_FENTA and BF-FENTA outperformed the acyclic chelator CHX-A”-DTPA-NHMe in serum and DTPA challenge experiments. While the [^177^Lu]Lu^3+^ complex H_4_FENTA appeared to match the inertness of DOTA in serum, a DTPA challenge study revealed that DOTA retained [^177^Lu]Lu^3+^ to a greater extent. Additionally, replacing one carboxylic acid of H_4_FENTA with an amide in BF-FENTA resulted in reduced kinetic inertness.

With [^213^Bi]Bi^3+^, H_4_FENTA achieved efficient radiolabeling at very high AMAs (~ 200 MBq/nmol) within 5 min. Additionally, it showed pronounced selectivity for [^213^Bi]Bi^3+^ in the presence of an excess of competing metal ions. Although BF-FENTA also exhibited rapid incorporation of [^213^Bi]Bi^3+^, its RCCs at high AMAs were lower, and its selectivity in the presence of Fe^3+^ was reduced compared to H_4_FENTA. Regarding the kinetic inertness, both [^213^Bi]Bi^3+^ complexes of H_4_FENTA and BF-FENTA remained intact over 90 min in both buffer at RT and human serum at 37 °C, like DOTA and CHX-A”-DTPA-NHMe. Although structural improvements for the bifunctional derivative are suggested, H_4_FENTA appears to be a promising chelator for bismuth-213 as it shows similar stability in vitro compared to DOTA, while providing fast radiolabeling under mild conditions.

## Materials and methods

### General

Reagents used for synthesis were purchased from commercial sources (BLDPharm, J&K Scientific, Merck, Acros Organics, TCI Chemicals, or Fluorochem). All (dry) solvents used for synthesis and purification were purchased from Merck, Thermo Fisher Scientific, or VWR International. Thin layer chromatography (TLC) was performed using silica gel 60 F254 (Merck), neutral alumina 60 F254 (Fisher scientific), and silica gel 60 RP-18 F254 (Merck). 60–200 Mesh silica gel (Acros) was used as stationary phase for manual column chromatography. Flash chromatography was performed on a CombiFlash EZ Prep using BGB Scorpius Alumina Neutral 55 Å, irregular 60 µm, 8 g cartridges. Preparative HPLC was carried out on a Waters preparative HPLC system, equipped with a Waters 2487 dual wavelength UV/Vis detector and a Waters SQ Detector 2 mass spectrometer, using an XBridge BEH C18 OBD prep column (130 Å, 5 µm, 19 mm × 150 mm). 

 Nuclear magnetic resonance (NMR) spectra were recorded on a Bruker Avance III HD 400 spectrometer (400 MHz for 1 H NMR and 101 MHz for 13C NMR) or on a Bruker Avance II + 600 spectrometer (600 MHz for 1 H NMR and 151 MHz for 13C NMR). The chemical shifts (δ, ppm) were determined relative to the internal solvent signal. High-resolution mass spectroscopy (HRMS) data were acquired on a quadrupole orthogonal acceleration time-of-flight mass spectrometer (Synapt G2 HDMS, Waters, Milford, MA). Samples were infused at 3 μL min − 1 and the spectrum was obtained in positive ionization mode with a resolution of 15 000 (FWHM) using leucine enkephalin as lock mass.

All labeling reactions were carried out in 1.5 mL low protein binding screw-top microtubes (Sarstedt, Belgium). Labeling buffers were prepared with trace metal grade chemicals and treated overnight with Chelex® 100 resin (50–100 mesh, sodium form). Instant thin layer chromatography (iTLC) was performed using glass microfiber chromatography paper strips impregnated with silica gel (iTLC-SG, Agilent Technologies, Belgium).

The iTLC strips were analyzed using an automated gamma counter (2470Wizard^2^, Perkin Elmer, Belgium), a TLC scanner (miniGITA, Elysia-Raytest®, Germany), or phosphor imaging autoradiography (CR-35 Bio, Elysia-Raytest®, Germany). The latter two instruments allowed the visualization of the iTLC profiles for qualitative analysis, enabling the iTLC strips to be cut at the correct position. Radio-HPLC analyses were performed on a Waters Acquity Arc system equipped with a GABI Nova radio detector (Elysia-Raytest) using a XBridge Peptide BEH C18 column (130 Å, 5 µm, 4.6 mm × 150 mm).

### Radionuclide sources and preparation

[^161^Tb]TbCl_3_ used in this study was produced at SCK CEN, either through in-house production or via the manufacturing agreement with TerThera. High radionuclidic purity (Tb-161 ≥ 99.9%, Tb-160 ≤ 0.01%, sum of other impurities ≤ 0.01%) and chemical purity (Fe: < 0.15 µg/GBq, Cu: < 0.1 µg/GBq, Zn: < 0.25 µg/GBq, Pb: < 0.1 µg/GBq, Gd-160: < 0.1 µg/GBq) was confirmed using standardized QC methods, including ICP-MS, gamma spectrometry, and DOTATATE radiolabeling at 200 MBq/nmol. [^177^Lu]LuCl_3_ was purchased from ITM Isotope Technologies Munich SE. High radionuclidic purity (Yb-175 ≤ 0.01%, sum of other impurities ≤ 0.01%) and chemical purity (Fe: < 0.25 µg/GBq, Cu: < 0.5 µg/GBq, Zn: < 0.5 µg/GBq, Pb: < 0.5 µg/GBq, Yb-176: < 0.14 µg/GBq, sum of impurities ≤ 0.5 µg/GBq) was reported in the certificate of analysis. Stock solutions of [^161^Tb]TbCl_3_ and [^177^Lu]LuCl_3_ were diluted with a 50 mM HCl solution prepared with trace-metal grade water to obtain the desired activity concentrations.

[^213^Bi]Bi^3+^ was obtained from an in-house prepared actinium-225/bismuth-213 generator. A high-density polyethylene tubing with an inner diameter of 4 mm and a length of 80 mm served as the Ac/Bi separation column. It was equipped with a 30 mikron PTFE frit (Instrument Solutions, The Netherlands) and loaded with an aqueous slurry of AG-MP-50 resin (100–200 mesh, H^+^ form, Eichrom, USA) to yield a total bed height of 10 mm. A second PTFE frit was used to fix the resin bed. The column was connected to 1/16″ PTFE tubing using two PEEK male luer to 10–32 female thread adapters (P656, VWR, Belgium). A peristaltic pump (Reglo Digital, Ismatec, Germany) equipped with a 0.64 mm inner diameter SC0306A tubing (VWR, Belgium) served to pump solution over the column at a flow rate of 0.5 mL/min. A purified [^225^Ac]Ac^3+^ solution was loaded on the column at a flow rate of 0.5 ml/min. Additional 5 mL of 0.05 M HNO_3_ was used to rinse the tubing and column for a quantitative loading. Subsequently, the column was washed with 5 mL of deionized water and then conditioned with 2 mL of 0.1 M NaI/0.1 M HCl solution.

A freshly made 0.1 M NaI/HCl solution was used as eluent to milk [^213^Bi]Bi^3+^ as [BiI_4_]^−^ and [BiI_5_]^2−^. Between elutions, the generator was allowed to stand for at least 3 h. All activities with [^213^Bi]Bi^3+^ reported in this work were determined within one minute post elution and all labeling experiments with [^213^Bi]Bi^3+^ were performed within 2–5 min post elution.

The purified [^225^Ac]Ac^3+^ was obtained via two in-house thorium-229 generators. They were milked using a tandem of a TEVA/ branched DGA 2 mL separation columns (TE-R10-S and DB-R10-S, respectively) and a vacuum box system (all from Triskem, France) to obtain actinium-225 and radium-225 fractions for further processing. The actinium-225 solution, present in 0.05 M HNO_3_, was subsequently evaporated and adjusted to 4 M HNO_3_ to undergo a second purification for the complete removal of residual thorium-229. The radium-225 fraction, already present in 4 M HNO_3_, was stored for 2 – 3 weeks and underwent a second purification to yield additional actinium-225. In each case, the final actinium-225 product was eluted in 5 mL of 0.05 M HNO_3_ and its activity measured using a CRC-55tR dose calibrator (Mirion, USA) at least 5 h after elution to allow for the actinium-225 decay chain to reestablish equilibrium.

### Synthesis

2,9-Bis(chloromethyl)-1,10-phenanthroline **1**, 2-(2-azidoethoxy)ethanamine **4**, and *N*-nosylglycine **5** were synthesized according to literature procedures (Weijnen and Engbersen 1993b; Chandler et al. 1981; Azagarsamy et al. 2010; Tovstiga et al. 2014).

#### Tetra-tert-butyl 2,2′,2″,2‴-(((1,10-phenanthroline-2,9-diyl)bis(methylene))bis(azanetriyl))tetra-acetate (3)

A modified literature procedure was used for the synthesis of **3** (Mukkala et al. 1992).

2,9-Bis(chloromethyl)-1,10-phenanthroline **1** (150 mg, 0.54 mmol, 1 equiv.) was added to a solution of di-*tert*-butyl iminodiacetate **2** (266 mg, 1.08 mmol, 2 equiv.) and DIPEA (0.28 ml, 1.62 mmol, 3 equiv.) in 5 ml anhydrous DMF. The mixture was stirred for 18 h at 85 °C under Ar atmosphere. After complete conversion, DMF was azeotropically removed with toluene *in vacuo*. The crude residue was dissolved in CHCl_3_, washed with 1 M HCl and dried over Na_2_SO_4_ and concentrated *in vacuo*. The desired product **3** was obtained after neutral alumina flash column chromatography (100% CHCl_3_ to 95:5 CHCl_3_: iPrOH) in 76% yield (286,9 mg) as an orange oil.

^1^H NMR (600 MHz, Chloroform-*d*) δ 8.23–8.19 (m, 4H), 7.73 (s, 2H), 4.44 (s, 4H), 3.54 (s, 8H), 1.46 (s, 36H) (Fig. S16).

^13^C NMR (101 MHz, Chloroform-*d*) δ 170.8, 161.3, 145.4, 136.9, 128.0, 126.0, 122.2, 81.2, 61.2, 56.2, 28.3 (Fig. S17).

HRMS (ESI^+^): *m/z* calculated for C_38_H_54_N_4_O_8_ [M + H]^+^: 695.4014; found: 695.4006.

#### 2,2′,2″,2‴-(((1,10-Phenanthroline-2,9-diyl)bis(methylene))bis(azanetriyl))tetraacetic acid (H_4_FENTA)

A modified literature procedure was used for the synthesis of **H**_**4**_**FENTA** (Mukkala et al. 1992).

TFA (1.2 mL, 15.96 mmol, 118 equiv.) was added to a solution of **4** (94 mg, 0.14 mmol, 1 equiv.) in DCM (1.3 mL) at 0 °C and the mixture was stirred for 3 h RT. The solvent was removed *in vacuo*, after which the residue was dissolved in H_2_O and purified by RP-HPLC (Mobile phase: (A) ACN with 0.1% formic acid, (B) H_2_O with 0.1% formic acid; Gradient: 5% A (0–2 min), 5–40% A (2–15 min); Flow: 20 mL/min; t_r_ = 4.5 min) to yield H_4_FENTA (29 mg, 46%) as a white solid.

^1^H NMR (600 MHz, DMSO-*d*_6_) δ 8.51 (d, *J* = 8.3 Hz, 2H), 8.01 (d, *J* = 8.3 Hz, 2H), 7.97 (s, 2H), 4.33 (s, 4H), 3.59 (s, 8H) (Fig. S18).

^13^C NMR (151 MHz, DMSO-*d*_6_) δ 172.5, 160.0, 137.2, 127.7, 126.1, 122.5, 59.8, 54.7 (Fig. S19).

HRMS (ESI^−^): *m/z* calculated for C_22_H_22_N_4_O_8_ [M—H]^−^: 469.13647; found: 469.1360.

#### N-(2-(2-Azidoethoxy)ethyl)-2-((2-nitrophenyl)sulfonamido)acetamide (6)

To a solution of *N*-nosylglycine **4** (500 mg, 1.91 mmol, 1 equiv.) in 1 ml anhydrous DMF was added DPPA (0.621 ml, 2.88 mmol, 1.5 equiv.) at 0 °C, followed by TEA (0.534 ml, 3.84 mmol, 2.0 equiv.). The solution was stirred at 0 °C for 20 min under N_2_ atmosphere. To the mixture, a solution of 2-(2-azidoethoxy)ethan-1-amine **5** (400.1 mg, 3.07 mmol, 1.6 equiv.) in 1 ml anhydrous DMF was added dropwise over 1 min. The reaction was stirred at RT for 18 h. DMF was then azeotropically removed with toluene *in vacuo*. The residue was purified by silica gel column chromatography (1% MeOH in DCM). The title compound **6** was obtained as a yellow oil in 73% yield (524 mg).

^1^H NMR (600 MHz, Chloroform-*d*) δ 8.12–8.09 (m, 1H), 7.91–7.89 (m, 1H), 7.78–7.74 (m, 2H), 6.62 (broad t, *J* = 5.0 Hz, 1H), 3.77 (s, 2H), 3.66 (t, *J* = 4.8 Hz, 2H), 3.54 (t, *J* = 5.1 Hz, 2H), 3.46–3.43 (m, 2H), 3.37 (t, *J* = 4.8 Hz, 2H) (Fig. S20).

^13^C NMR (101 MHz, Chloroform-*d*) δ 167.5, 148.1, 134.2, 133.1, 133.0, 131.2, 125.8, 70.3, 69.5, 51.7, 46.3, 39.5 (Fig. S21).

HRMS (ESI^+^): *m/z* calculated for C_18_H_26_N_6_O_8_S [M + H]^+^: 373.0925; found: 373.0930.

#### Tert-butyl N-(2-((2-(2-azidoethoxy)ethyl)amino)-2-oxoethyl)-N-((2-nitrophenyl)sulfonyl)glycinate (8)

**6** (390 mg, 3.14 mmol, 1 equiv.) was dissolved in 5 ml of anhydrous ACN. Tert-butyl bromoacetate (0.464 ml, 3.14 mmol, 1 equiv.) and DIPEA (0.547 ml, 3.14 mmol, 1 equiv.) were added and the mixture was stirred for 17 h at reflux, under N_2_ atmosphere. The solvent was removed *in vacuo* and the orange oily residue was redissolved in EtOAc (~ 40 ml), washed with 1 N HCl (4 × 10 ml) and brine. The organic layer was dried over Na_2_SO_4_, filtered and evaporated. The crude residue was packed on celite and purified by silica gel column chromatography (50:50 PE:EtOAc to 20:80 PE:EtOAc). The desired compound **8** was obtained in 84% yield (426 mg) as a yellow oil.

^1^H NMR (600 MHz, Chloroform-*d*) δ 8.10–8.07 (m, 1H), 7.75–7.70 (m, 2H), 7.68–7.65 (m, 1H), 7.08 (t, *J* = 5.3 Hz, 1H), 3.64 (t, *J* = 4.9 Hz, 2H), 3.53 (t, *J* = 5.1 Hz, 2H), 3.45–3.41 (m, 2H), 3.38 (t, *J* = 5.1 Hz, 2H), 1.40 (s, 9H) (Fig. S22).

^13^C NMR (151 MHz, Chloroform-*d*) δ 167.8, 167.6, 148.1, 134.3, 132.4, 132.2, 131.5, 124.6, 83.2, 70.1, 69.5, 52.2, 50.9, 50.8, 39.4, 28.0 (Fig. S23).

HRMS (ESI^+^): *m/z* calculated for C_18_H_26_N_6_O_8_S [M + H]^+^: 487.1605; found: 487.1607.

#### Tert-butyl (2-((2-(2-azidoethoxy)ethyl)amino)-2-oxoethyl)glycinate (9)

**8** (382 mg, 0.785 mmol, 1.0 equiv.) was dissolved in THF. Thiophenol (0.16 ml, 1.57 mmol, 2.0 equiv.) and K_2_CO_3_ (542.6 mg, 3.926 mmol, 5.0 equiv.) were added and the mixture was stirred for 18 h at RT. The mixture was diluted with THF, centrifuged and the organic layer was removed (repeated 3 times). The combined organic layers were concentrated *in vacuo* and the crude product was purified by silica gel column chromatography (100% DCM to 95:5 DCM:MeOH). The desired compound **9** was obtained as a yellow oil in 93% yield (219.6 mg).

^1^H NMR (400 MHz, Chloroform-*d*) δ 7.54 (broad s, 1H), 3.69–3.64 (m, 2H), 3.60–3.56 (m, 2H), 3.53–3.47 (m, 2H), 3.41–3.35 (m, 2H), 3.30 (s, 2H), 3.28 (s, 2H), 1.46 (s, 9H) (Fig. S24).

^13^C NMR (151 MHz, CDCl_3_) δ 171.5, 171.3, 81.6, 69.94, 69.91, 52.3, 51.7, 50.7, 38.7, 28.1 (Fig. S25).

HRMS (ESI^+^): *m/z* calculated for C_12_H_23_N_5_O_4_ [M + H]^+^: 302.1823; found: 302.1822.

#### Di-tert-butyl 2,2′-(((9-(chloromethyl)-1,10-phenanthrolin-2-yl)methyl)azanediyl)diacetate (10)

**2** (25 mg, 0.10 mmol, 1 equiv.) was dissolved in 2 mL anhydrous ACN. **1** (57 mg, 0.21 mmol, 2 equiv.) and Na_2_CO_3_ (23 mg, 0.22 mmol, 2.1 equiv.) were added. The reaction mixture was stirred overnight at reflux under Ar atmosphere. The mixture was diluted with CHCl_3_, centrifuged and the organic layer was removed (repeated 3 times). The combined organic layers were concentrated *in vacuo*. The residue was filtered over silica (1% MeOH in CHCl_3_) and purified by silica gel column chromatography (100% CHCl_3_ to 99:1 CHCl_3_:MeOH). The title compound was obtained as an orange oil in 85% yield (43 mg).

^1^H NMR (600 MHz, Chloroform-*d*) δ 8.28–8.20 (m, 3H), 7.87 (m, 1H), 7.79–7.72 (m, 2H), 5.06 (s, 2H), 4.45 (s, 2H), 3.54 (s, 4H), 1.44 (s, 18H) (Fig. S26).

^13^C NMR (151 MHz, Chloroform-*d*) δ 170.6, 161.5, 157.2, 145.0, 144.8, 137.4, 137.2, 128.20, 128.15, 127.0, 125.7, 122.7, 122.3, 81.3, 61.0, 56.2, 47.7, 28.3 (Fig. S27).

HRMS (ESI^+^): *m/z* calculated for C_26_H_32_ClN_3_O_4_ [M + H]^+^: 486.2154; found: 486.2149.

#### Di-tert-butyl 2,2′-(((9-(((2-((2-(2-azidoethoxy)ethyl)amino)-2-oxoethyl)(2-(tert-butoxy)-2-oxoethyl)amino)methyl)-1,10-phenanthrolin-2-yl)methyl)azanediyl)diacetate (11)

**9** (31 mg, 0.06 mmol, 1 equiv.) was dissolved in 0.3 mL anhydrous ACN. Phenanthroline **10** (24 mg, 0.08 mmol, 1.3 equiv.) and Na_2_CO_3_ (8 mg, 0.09 mmol, 1.5 equiv.) were added and the mixture was stirred at reflux for 5 h under Ar atmosphere. The mixture was diluted with CHCl_3_, centrifuged and the organic layer was removed (repeated 3 times). The combined organic layers were concentrated *in vacuo* and the crude product was purified by neutral alumina flash column chromatography (100% CHCl_3_ to 92:8 CHCl_3_:iPrOH) in 79% yield (37 mg) as an orange oil.

^1^H NMR (600 MHz, Chloroform-*d*) δ 8.49 (s, 1H), 8.24 (d, *J* = 8.4 Hz, 2H), 8.19 (d, *J* = 8.3 Hz, 1H), 7.80 (d, *J* = 8.2 Hz, 1H), 7.79–7.74 (m, 2H), 4.43 (s, 2H), 4.33 (s, 2H), 3.55–3.50 (m, 10H), 3.44 (d, *J* = 4.3 Hz, 4H), 3.24 (t, *J* = 5.1 Hz, 2H), 1.47–1.45 (m, 27H) (Fig. S28).

^13^C NMR (151 MHz, Chloroform-*d*) δ 171.3, 170.8, 170.7, 161.2, 159.0, 145.7, 145.3, 137.1, 136.9, 128.2, 128.1, 126.6, 125.9, 122.7, 122.3, 81.7, 81.3, 69.9, 69.6, 61.3, 61.1, 58.8, 57.1, 56.1, 50.7, 38.9, 28.32, 28.28 (Fig. S29).

HRMS (ESI^+^): *m/z* calculated for C_35_H_54_N_8_O_8_ [M + H]^+^: 751.4137; found: 751.4133.

#### 2,2'-(((9-(((2-((2-(2-Azidoethoxy)ethyl)amino)-2-oxoethyl)(carboxymethyl)amino)methyl)-1,10-phenanthrolin-2-yl)methyl)azanediyl)diacetic acid (BF-FENTA)

TFA (0.9 mL) was added to a solution of **11** (65 mg, 0.09 mmol, 1 equiv.) in DCM (0.9 mL) at 0 °C and the mixture was stirred for 3 h at RT. The solvent was removed *in vacuo*, the residue was dissolved in H_2_O and purified by RP-HPLC (Mobile phase: (A) ACN with 0.1% formic acid, (B) H_2_O with 0.1% formic acid; Gradient: 5% A (0–2 min), 5–40% A (2–15 min); Flow: 20 mL/min; t_r_ = 10.5 min) to yield BF-FENTA (24 mg, 48%) as a white solid.

^1^H NMR (600 MHz, D_2_O) δ 8.88 (d, *J* = 8.4 Hz, 1H), 8.64 (d, *J* = 8.4 Hz, 1H), 8.14 (d, *J* = 8.3 Hz, 1H), 8.11–8.04 (m, 2H), 7.95 (d, *J* = 8.4 Hz, 1H), 5.06 (s, 2H), 4.83 (s, 2H), 4.13 (s, 4H), 3.99 (d, *J* = 7.2 Hz, 4H), 3.45 (t, *J* = 4.7 Hz, 2H), 3.34 (t, *J* = 5.3 Hz, 2H), 3.26 (t, *J* = 5.0 Hz, 3H), 3.23 (t, *J* = 5.3 Hz, 3H) (Fig. S30).

^13^C NMR (101 MHz, D_2_O) δ 173.1, 171.5, 169.6, 154.2, 153.2, 143.3, 141.2, 137.3, 136.9, 129.3, 129.1, 128.0, 126.9, 124.7, 69.0, 68.4, 57.0, 56.9, 56.6, 50.0, 38.8 (Fig. S31).

HRMS (ESI^+^): *m/z* calculated for C_26_H_30_N_8_O_8_ [M + H]^+^: 583.2259; found: 583.2269.

### Molar activity escalation study with terbium-161

[^161^Tb]Tb^3+^ (10 MBq, 10 µL) was added to a solution of H_4_FENTA, BF-FENTA, or DOTA (67—200 pmol, 40 µL) in NaOAc buffer (0.25 M, pH 4.7), along with AA (50 mM, 10 µL) dissolved in the same buffer. Each labeling reaction was shaken for 15 min at 40 °C. The RCC was determined by iTLC using ACN/H_2_O (3:1) as the mobile phase. Under these conditions, free [^161^Tb]Tb^3+^ remained at the baseline, while the chelated species migrated. 0.1 M citrate buffer (pH 4.8) was not a suitable eluent (Fig. S3). The iTLC strips were visualized by a miniGITA TLC scanner, cut in two halves at the appropriate height (Fig. S1) and quantified using a gamma counter. All experiments were performed in triplicate.

### Stability in buffer and serum, and DTPA challenge with terbium-161 and lutetium-177

[^161^Tb]Tb^3+^, or [^177^Lu]Lu^3+^ (75 MBq, 15 µL) was added to a solution of H_4_FENTA, BF-FENTA, CHX-A”-DTPA-NHMe or DOTA (2 nmol, 60 µL) in NaOAc buffer (0.25 M, pH 4.7), along with AA (200 mM, 15 µL) dissolved in the same buffer. Each labeling reaction was shaken for 15 min at 40 °C, except for the reactions with DOTA (15 min, 85 °C). Subsequently, the solution was divided equally (30 µL) into three 1.5 mL low protein binding screw-top microtubes (Sarstedt, Belgium).

To the first vial, 70 µL of NaOAc buffer (0.25 M, pH 4.7) was added, and the solution was kept at RT to monitor the stability in buffer over 7 days. To the second vial, 90 µL of human serum was added, and the solution was incubated at 37 °C. The % radiometal bound under both conditions was determined by iTLC using ACN/H_2_O (3:1) as the mobile phase. The iTLC strips were visualized by a miniGITA TLC scanner, cut in two halves at the appropriate height (Figs. S1, S2) and quantified using a gamma counter. All experiments were performed in triplicate.

To the third vial, 20 µL of a DTPA solution (25 mM) in NaOAc buffer (0.25 M, pH 4.7) was added, followed by 130 µL of the same buffer. This mixture was kept at RT to monitor transchelation over 7 days. The radiochemical purity was determined by radio-HPLC (Figs. S4, S5) and iTLC using ACN/H_2_O (3:1) as the mobile phase.

### Association experiment with bismuth-213

[^213^Bi][BiI_4_]^−^/[^213^Bi][BiI_5_]^2−^ (1.3 -1.4 MBq, 40 µL) was added to a chelator solution (600 pmol, 10 µL) in NaOAc buffer (1 M, pH 4.8), along with AA (180 mM, 10 µL) dissolved in the same buffer. Each labeling reaction was shaken at 40 °C, and samples were taken at the given time points. The RCC was determined by iTLC using a 0.1 M citrate buffer (pH 4.8) as the mobile phase. Under these conditions, free [^213^Bi]Bi^3+^ migrated without retention (R_f_ = 1), while the chelated species did show retention (Fig. S12E–F). The iTLC strips were visualized by autoradiography, cut in two halves at the appropriate height and quantified using a gamma counter. All experiments were performed in triplicate.

### Molar activity escalation study with bismuth-213

[^213^Bi][BiI_4_]^−^/[^213^Bi][BiI_5_]^2−^ (1.1–1.2 MBq, 40 µL) was added to a solution of H_4_FENTA, BF-FENTA, CHX-A”-DTPA-NHMe, or DOTA (0.6–2000 pmol, 10 µL) in NaOAc buffer (1 M, pH 4.8), along with AA (180 mM, 10 µL) dissolved in the same buffer. Each labeling reaction was shaken for 5 min at 40 °C, except DOTA (30 min, 90 °C). The RCC was determined by iTLC using a 0.1 M citrate buffer (pH 4.8) as the mobile phase for H_4_FENTA and BF-FENTA (as mentioned above), and ACN/H_2_O (3:1) for CHX-A”-DTPA-NHMe and DOTA (Fig. S12C, D). The iTLC strips were visualized by autoradiography, cut in two halves at the appropriate height and quantified using a gamma counter. All experiments were performed in triplicate.

### Metal challenge with bismuth-213

Solutions (0.6 mM) of trace metal grade ZnCl_2_, CuCl_2_, FeCl_3_ and BiCl_3_ were prepared in 0.1 M HCl. Each metal solution (10 µL) was added separately to a mixture of H_4_FENTA or BF-FENTA (10 µL, 0.6 nmol) in NaOAc buffer (1 M, pH 4.8) and AA (180 mM, 10 µL) dissolved in the same buffer. Next, [^213^Bi][BiI_4_]^−^/[^213^Bi][BiI_5_]^2−^ (1.3–1.4 MBq, 40 µL) was added. Each labeling reaction was shaken for 5 min at 40 °C, except for the reactions with DOTA (90 °C, 30 min). The RCC was determined by iTLC using a 0.1 M citrate buffer (pH 4.8) as the mobile phase for H_4_FENTA and BF-FENTA (as mentioned above), and ACN/H_2_O (3:1) for CHX-A”-DTPA-NHMe and DOTA. The iTLC strips were visualized by autoradiography, cut in two halves at the appropriate height (Fig. S12C–F) and quantified using a gamma counter. All experiments were performed in triplicate.

### Stability study in buffer and serum with bismuth-213

[^213^Bi][BiI_4_]^−^/[^213^Bi][BiI_5_]^2−^ (2.1–2.5 MBq, 60 µL) was added to a solution of H_4_FENTA, BF-FENTA, CHX-A”-DTPA-NHMe, or DOTA (900 pmol, 15 µL) in NaOAc buffer (1 M, pH 4.8), along with AA (180 mM, 15 µL) dissolved in the same buffer. Each labeling reaction was shaken for 5 min at 40 °C, except for the reactions with DOTA (30 min, 90 °C). Subsequently, 30 µL of this solution was added to 30 µL of human serum in a 1.5 mL low protein binding screw-top microtube (Sarstedt, Belgium).

The labeling solution was kept at RT to monitor the stability in buffer over 90 min. The sample with serum was incubated for 90 min at 37 °C. The % radiometal bound was determined by iTLC using a 0.1 M citrate buffer (pH 4.8) as the mobile phase for H_4_FENTA and BF-FENTA, and ACN/H_2_O (3:1) for CHX-A”-DTPA-NHMe and DOTA. The iTLC strips were visualized by autoradiography, cut in two halves at the appropriate height (Fig. S12) and quantified using a gamma counter. All experiments were performed in triplicate.

## Supplementary Information

Supplementary Material 1.

## Data Availability

Additional radiolabeling (Figs. S13, S14) and stability data (Figs. S6, S7, S10, S11, S15), iTLC (Figs. S1–S3, S12) and HPLC data (Figs. S4, S5, S8, S9), and NMR spectra (Figs. S16–S31) are available in the Electronic Supplementary Information (ESI).

## Abbreviations

3p-C-NETA: 2,2'-((1-(4,7-Bis(carboxymethyl)-1,4,7-triazonan-1-yl)-5-(4-nitrophenyl)pentan-2-yl)azanediyl)diacetic acid
AA: Ascorbic acid
AAZTA: 6-Amino-6-methylperhydro-1,4-diazepinetetraacetic acid
DOTA: 1,4,7,10-Tetraazacyclododecane-1,4,7,10-tetraacetic acid
DPPA: Diphenylphosphoryl azide
DTPA: Diethylenetriaminepentaacetic acid
H_4_BATA: Benzodioxatetraaza-18-crown-6 tetraacetic acid
H_4_FENTA: 2,2ʹ,2″,2‴-(((1,10-Phenanthroline-2,9-diyl)bis(methylene))bis(azanetriyl))tetraacetic acid
HRMS: High-resolution mass spectroscopy
iTLC: Instant thin layer chromatography
AMA: Apparent molar activity
MES: 2-(*N*-Morpholino)ethanesulfonic acid
PYTA: 3,6,10,13-Tetraaza-1,8(2,6)-dipyridinacyclotetradecaphane-3,6,10,13-tetraacetic acid
RCC: Radiochemical conversion
RP-HPLC: Reversed-phase high-performance liquid chromatography
TFA: Trifluoroacetic acid
TRT: Targeted radionuclide therapy
