# Supplementary material for: Radiolabeling efficiency of FENTA chelators and stability of their terbium-161, lutetium-177 and bismuth-213 complexes
Source: EJNMMI Radiopharm Chem. 2026 Apr 7;11:36. doi: 10.1186/s41181-026-00438-y (PMC13187069; doi:10.1186/s41181-026-00438-y)
Supplement: Supplementary file 1 — Supplementary Material 1. [file 41181_2026_438_MOESM1_ESM.pdf]

Supporting Information

Radiolabeling efficiency of FENTA chelators and stability of their terbium-161, lutetium-177 and bismuth-213 complexes

Cédric Bonneux[1,2], Sunay Rodriguez Pérez[1], Stephan Heinitz[1], Veronique Bogaerts[1], Michiel Van de Voorde[1], Thomas Cardinaels[2], Maarten Ooms[1], Wim Dehaen[2*], Tomas Opsomer[1*]

[1]Institute for Nuclear Medical Applications, Belgian Nuclear Research Centre (SCK CEN), Mol, Belgium; [2]KU Leuven, Department of Chemistry, Sustainable Chemistry for Metals and Molecules, Leuven, Belgium

Email: wim.dehaen@kuleuven.be, tomas.opsomer@sckcen.be

Radiolabeling studies

Terbium-161 and lutetium-177

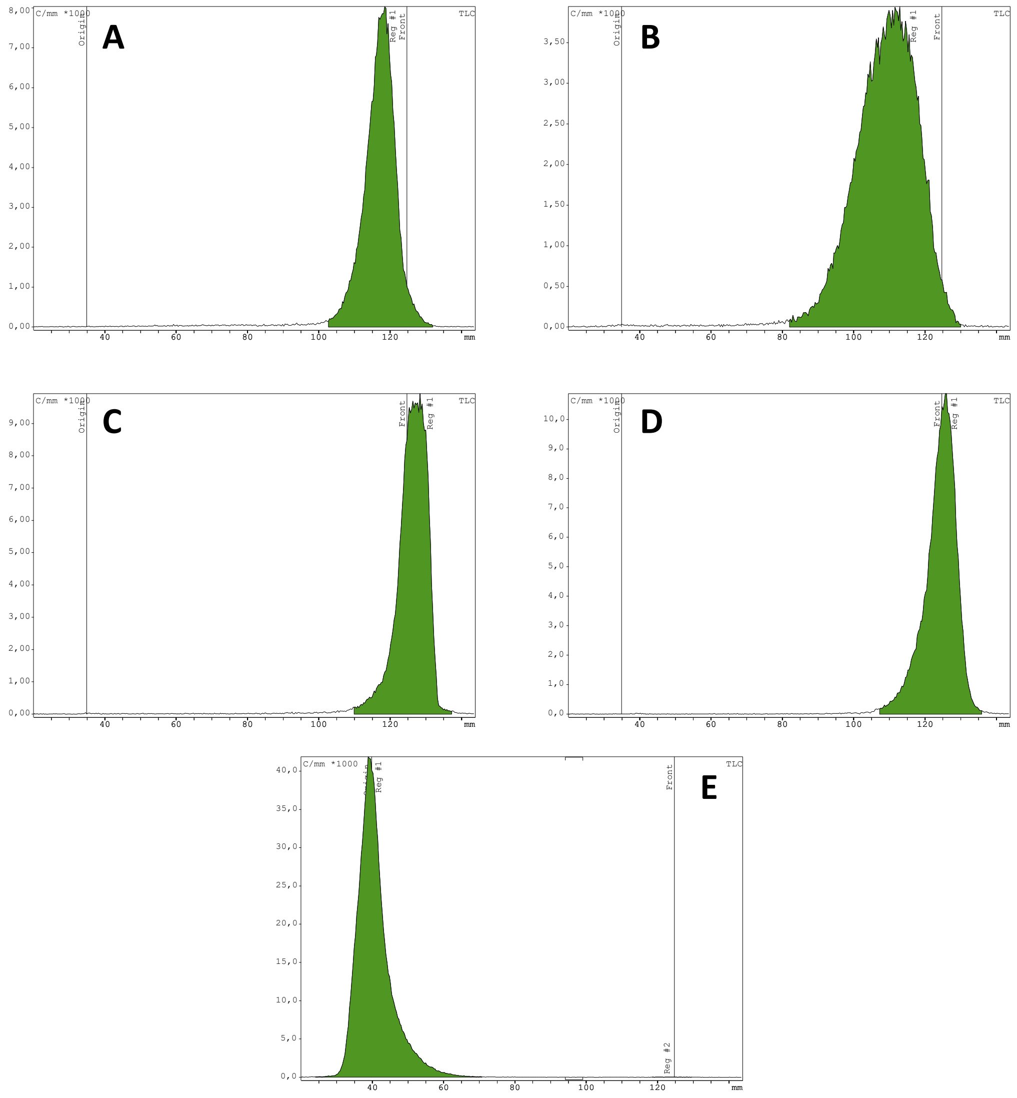

Fig. S1 iTLC chromatograms of [^{161}Tb]Tb-FENTA (A), [^{161}Tb]Tb-BF-FENTA (B), [^{161}Tb]Tb-CHX-A''-DTPA-NHMe (C), [^{161}Tb]Tb-DOTA (D), and free [^{161}Tb]Tb^{3+} (E). ACN:H_2O (3:1) was used as the mobile phase and visualization was performed with a miniGITA TLC scanner. TLC plates were cut in the middle before quantifying activity using a gamma counter.

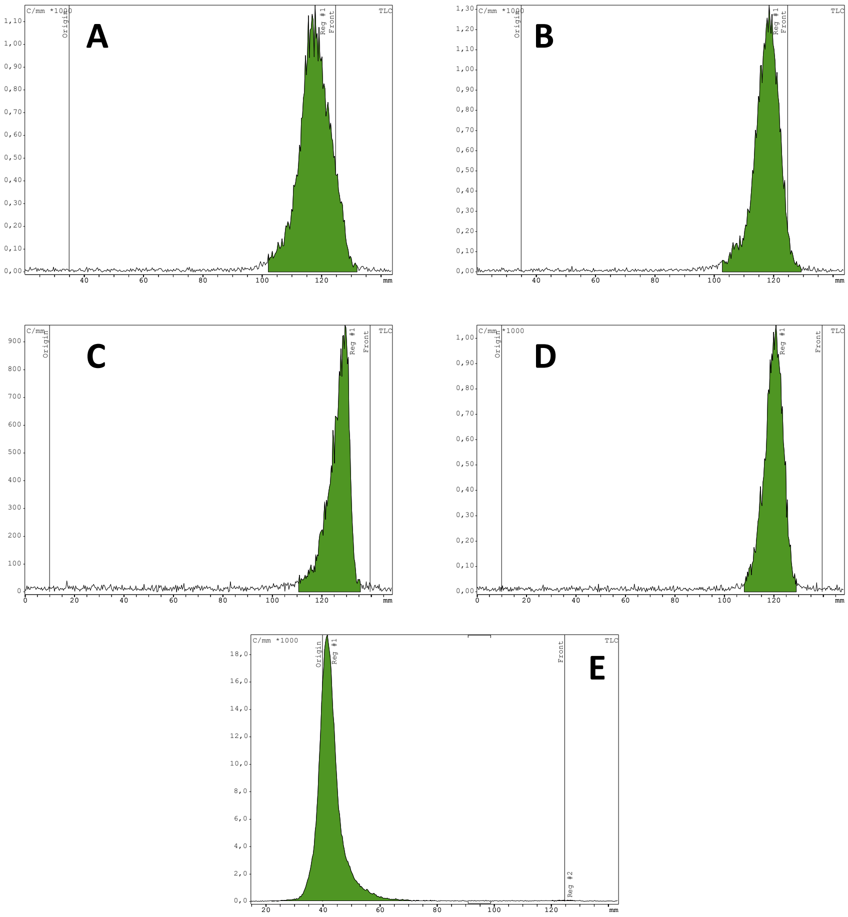

Fig. S2 iTLC chromatograms of [^{177}Lu]Lu-FENTA (A), [^{177}Lu]Lu-BF-FENTA (B), [^{177}Lu]Lu-CHX-A''-DTPA-NHMe (C), [^{177}Lu]Lu-DOTA (D), and free [^{177}Lu]Lu^{3+} (E). ACN:H_2O (3:1) was used as the mobile phase and visualization was performed with a miniGITA TLC scanner. TLC plates were cut in the middle before quantifying activity using a gamma counter.

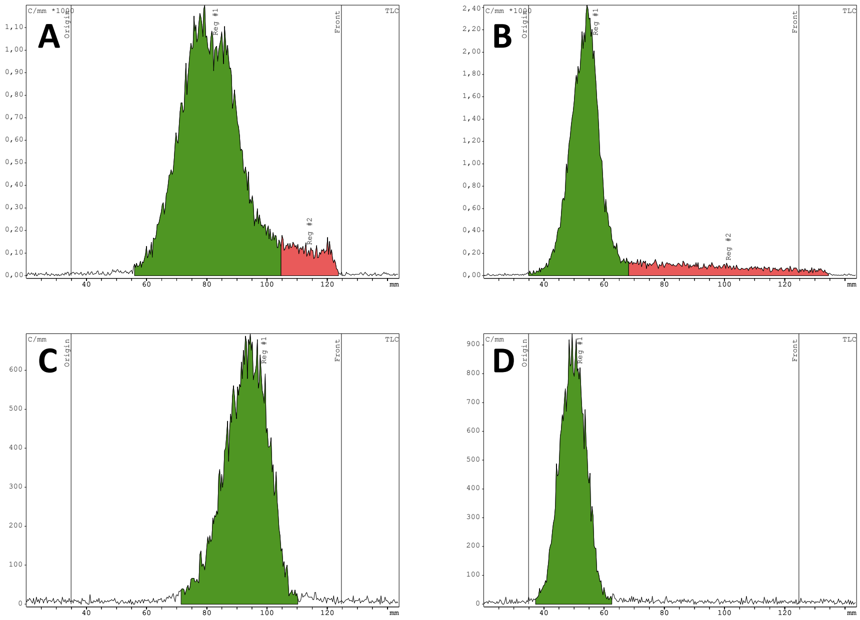

Fig. S3 iTLC chromatograms of [^{161}Tb]Tb-FENTA (A), [^{161}Tb]Tb-BF-FENTA (B), [^{177}Lu]Lu-FENTA (C), and [^{177}Lu]Lu-BF-FENTA (D). Citrate buffer (0.1 M, pH 4.8) was used as the mobile phase and visualization was performed with a miniGITA TLC scanner.

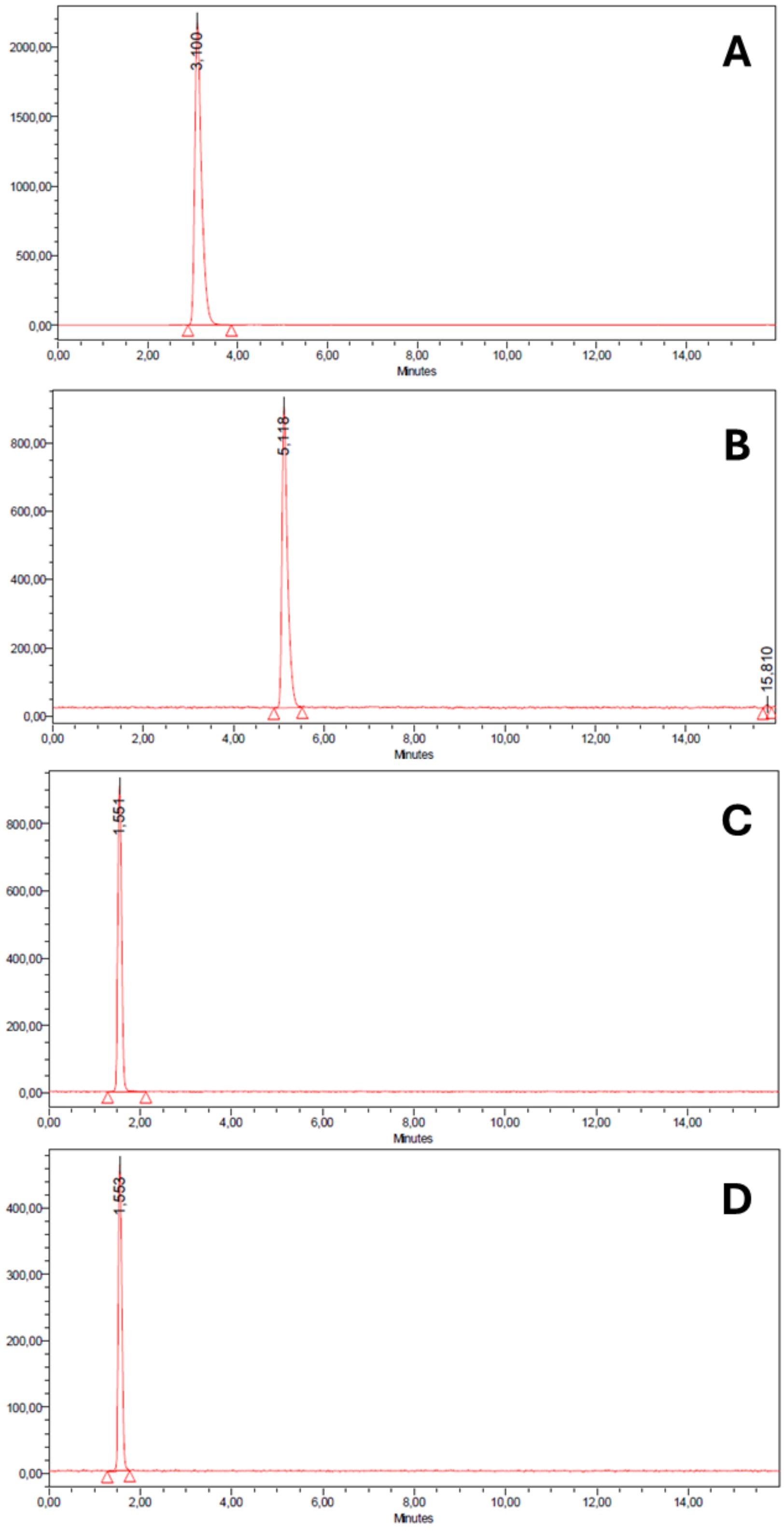

Fig. S4 Radio-HPLC chromatograms of [^{161}Tb]Tb-FENTA (A), [^{177}Lu]Lu-FENTA (B), [^{161}Tb]Tb-DTPA (C), and [^{177}Lu]Lu-DTPA (D). Mobile phase: (A) 10 mM NH_4OAc in ACN:H_2O (95:5), (B) 10 mM NH_4OAc in ACN:H_2O (5:95); Gradient: 5% A (0 – 1 min), 5 – 60 % A (1 – 10 min), 95 % A (10 – 13 min), 5 % A (13 – 16 min); Flow: 1 mL/min.

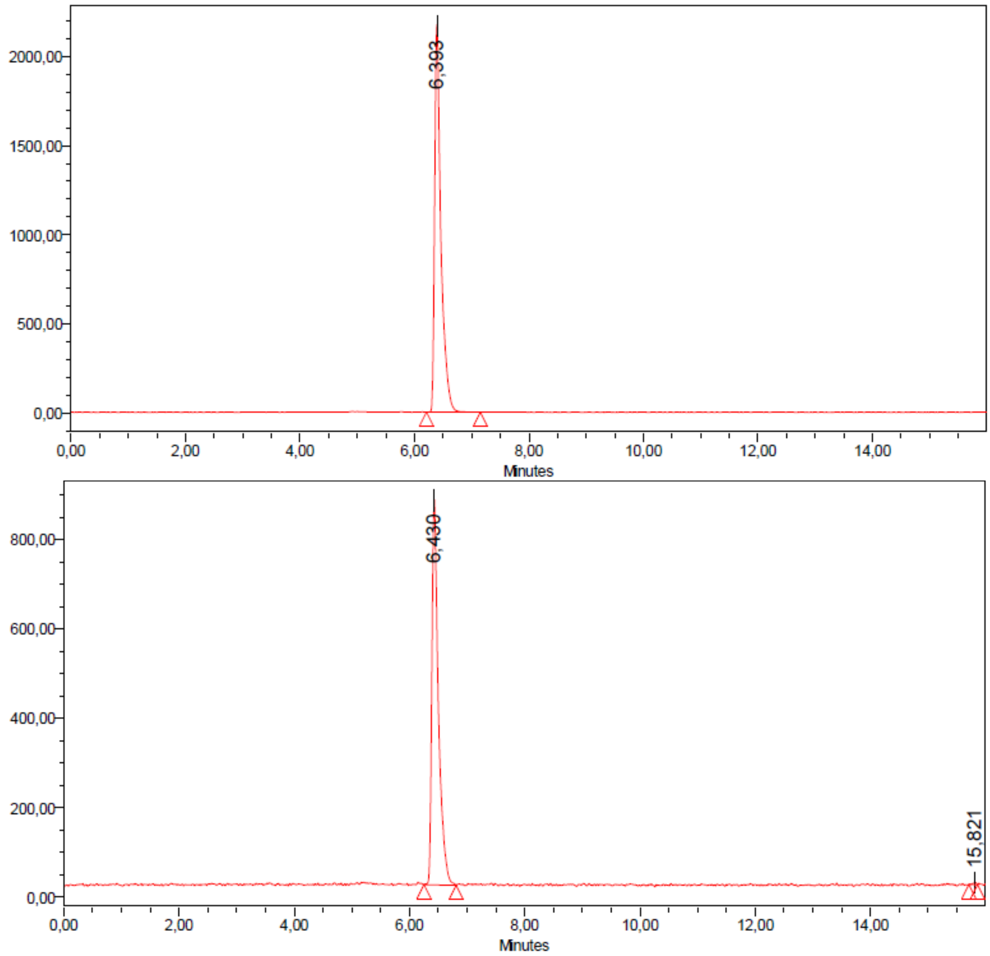

Fig. S5 Radio-HPLC chromatograms of [^{161}Tb]Tb-BF-FENTA (top) and [^{177}Lu]Lu-BF-FENTA (bot). Mobile phase: (A) 10 mM NH_4OAc in ACN:H_2O (95:5), (B) 10 mM NH_4OAc in ACN:H_2O (5:95); Gradient: 5% A (0 – 1 min), 5 – 40 % A (1 – 10 min), 95 % A (10 – 13 min), 5 % A (13 – 16 min); Flow: 1 mL/min.

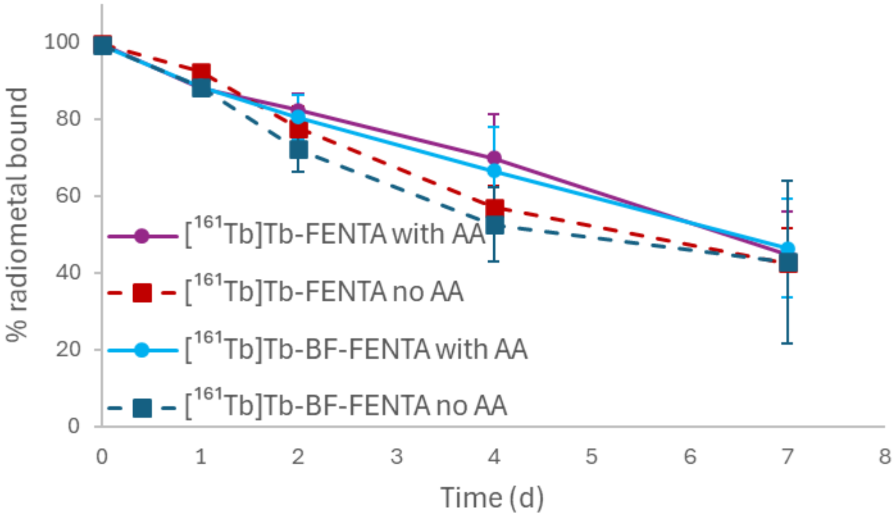

Fig. S6 Stability of $[^{161}Tb]Tb^{3+}$ complexes of H_4FENTA and BF-FENTA over time in acetate buffer (0.25 M, pH 4.7) at RT. All reactions were carried out in acetate buffer with 50 MBq $[^{161}Tb]Tb^{3+}$ at an AMA of 100 MBq/nmol in the presence or absence of AA (33mM), at 40°C for 15 min. The total volume was 60 µL. After radiolabeling, the solution was diluted 3.33-fold with acetate buffer. The percentage of radiometal bound was quantified using a gamma counter (cut and count method; n = 3).

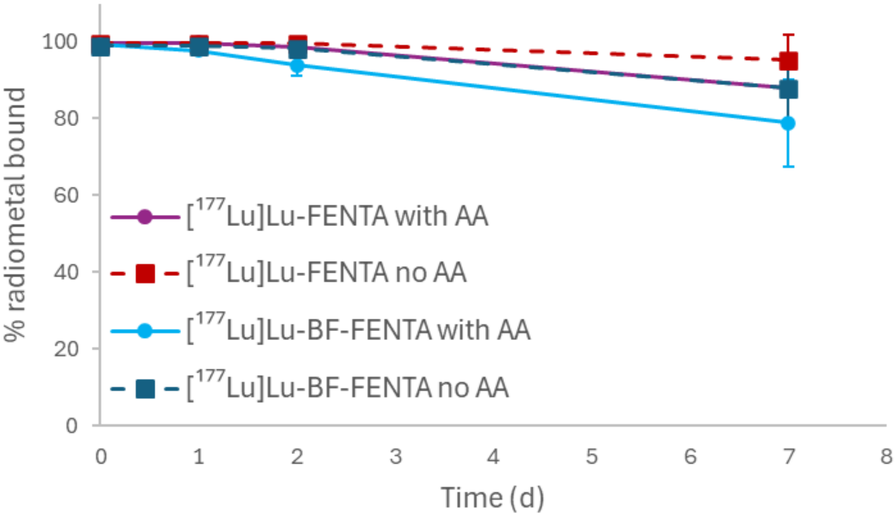

Fig. S7 Stability of $[^{177}Lu]Lu^{3+}$ complexes of H_4FENTA and BF-FENTA over time in acetate buffer (0.25 M, pH 4.7) at RT. All reactions were carried out in acetate buffer with 50 MBq $[^{177}Lu]Lu^{3+}$ at an AMA of 100 MBq/nmol in the presence or absence of AA (33mM), at 40°C for 15 min. The total volume was 60 µL. After radiolabeling, the solution was diluted 3.33-fold with acetate buffer. The percentage of radiometal bound was quantified using a gamma counter (cut and count method; n = 3).

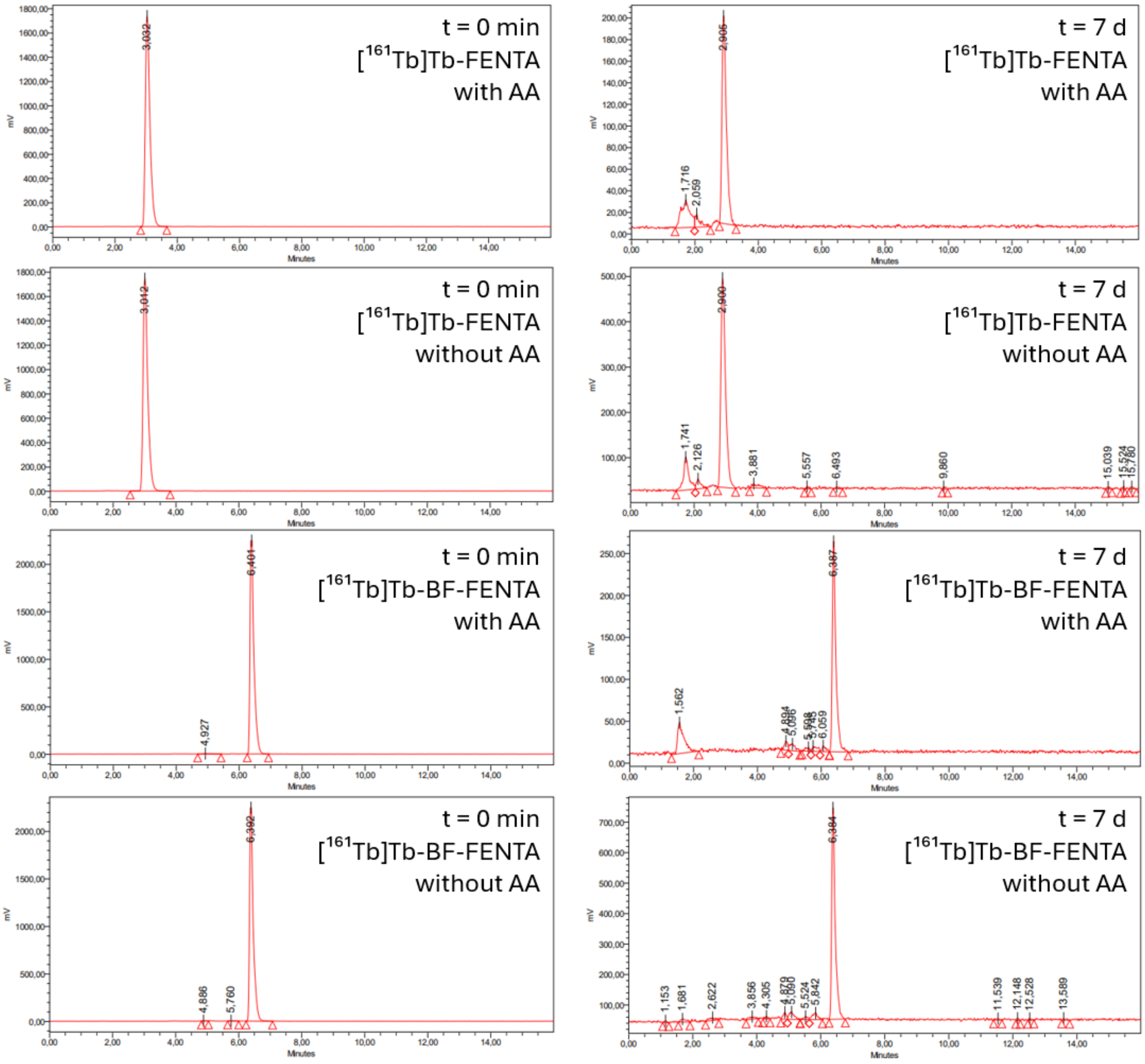

Fig. S8 Radio-HPLC chromatograms of $[^{161}Tb]Tb^{3+}$ complexes of H_4FENTA and BF-FENTA over time in acetate buffer (0.25 M, pH 4.7) at RT. All reactions were carried out in acetate buffer with 50 MBq $[^{161}Tb]Tb^{3+}$ at an AMA of 100 MBq/nmol in the presence or absence of AA (33mM), at 40°C for 15 min. The total volume was 60 µL. After radiolabeling, the solution was diluted 3.33-fold with acetate buffer. HPLC method: See **Fig. S4** and **S5**.

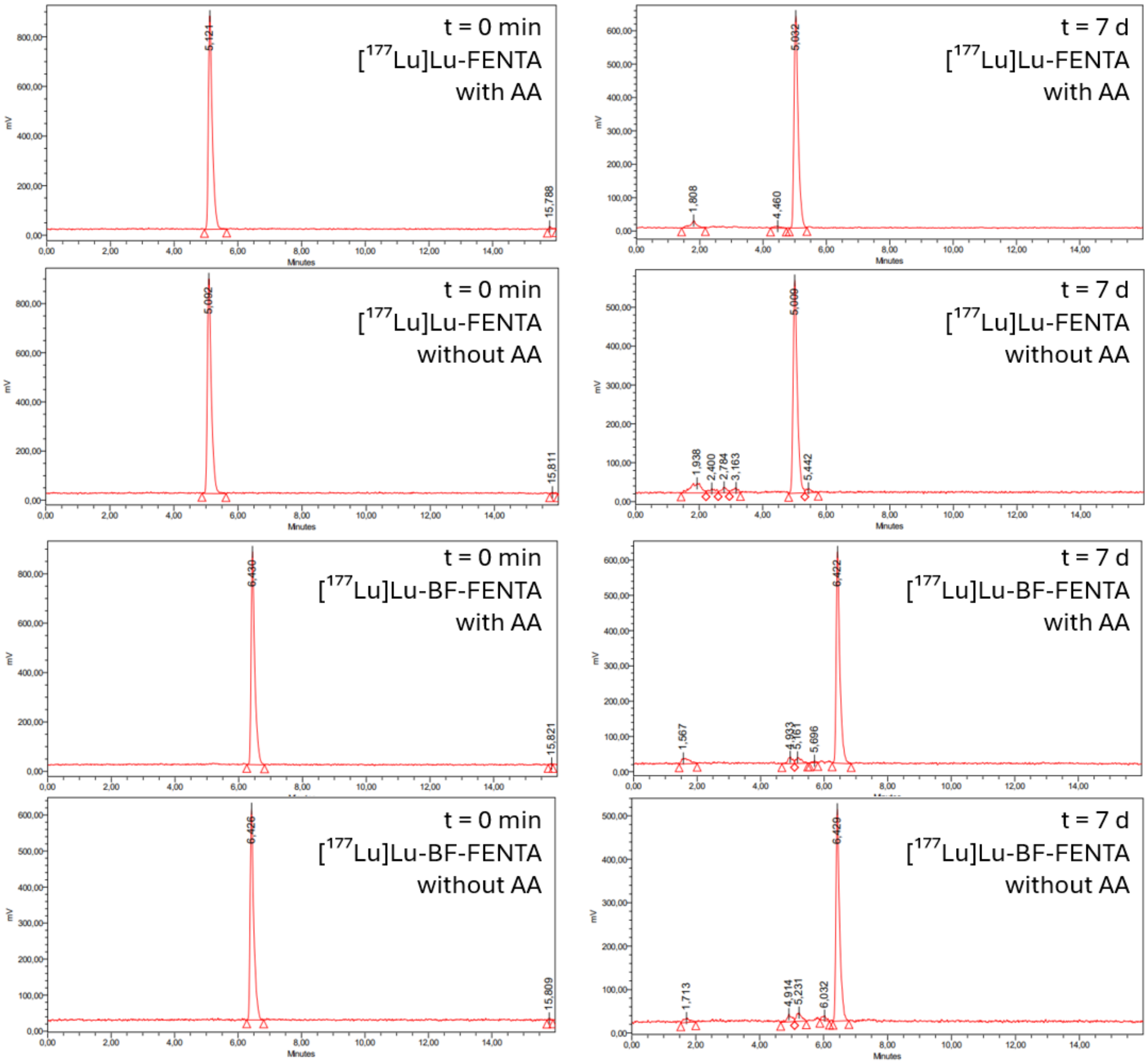

Fig. S9 Radio-HPLC chromatograms of $[^{177}Lu]Lu^{3+}$ complexes of H_4FENTA and BF-FENTA over time in acetate buffer (0.25 M, pH 4.7) at RT. All reactions were carried out in acetate buffer with 50 MBq $[^{177}Lu]Lu^{3+}$ at an AMA of 100 MBq/nmol in the presence or absence of AA (33mM), at 40°C for 15 min. The total volume was 60 µL. After radiolabeling, the solution was diluted 3.33-fold with acetate buffer. HPLC method: See **Fig. S4** and **S5**.

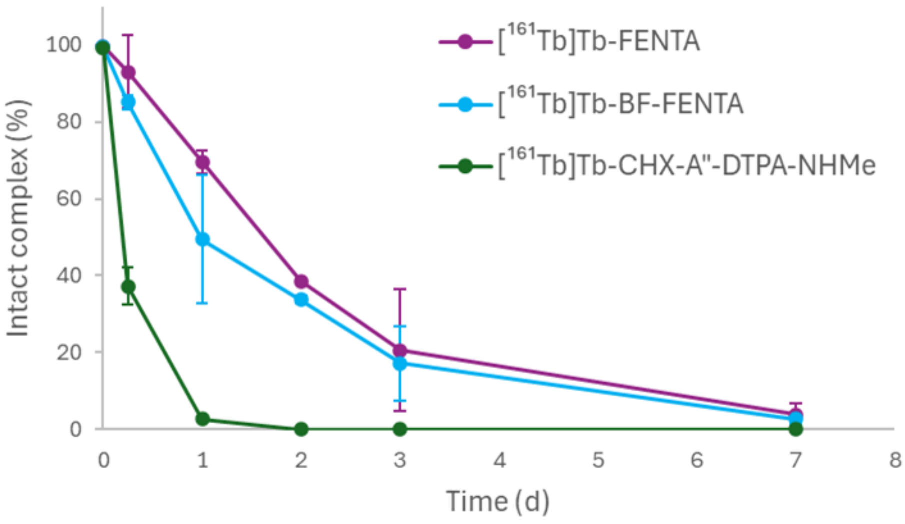

Fig. S10 DTPA challenge of [^{161}Tb]Tb^{3+} complexes of H_4FENTA, BF-FENTA and CHX-A"-DTPA in the presence of a 1000-fold DTPA (DTPA to ligand ratio). All reactions were carried out in acetate buffer (0.25 M, pH 4.7, 75 µL) with 75 MBq (15 µL) at an AMA of 50 MBq/nmol in the presence of AA (33mM), at 40°C for 15 min. After radiolabeling, the solution was diluted 6-fold with acetate buffer. The percentage of intact complex was monitored by radio-HPLC analysis (n = 2).

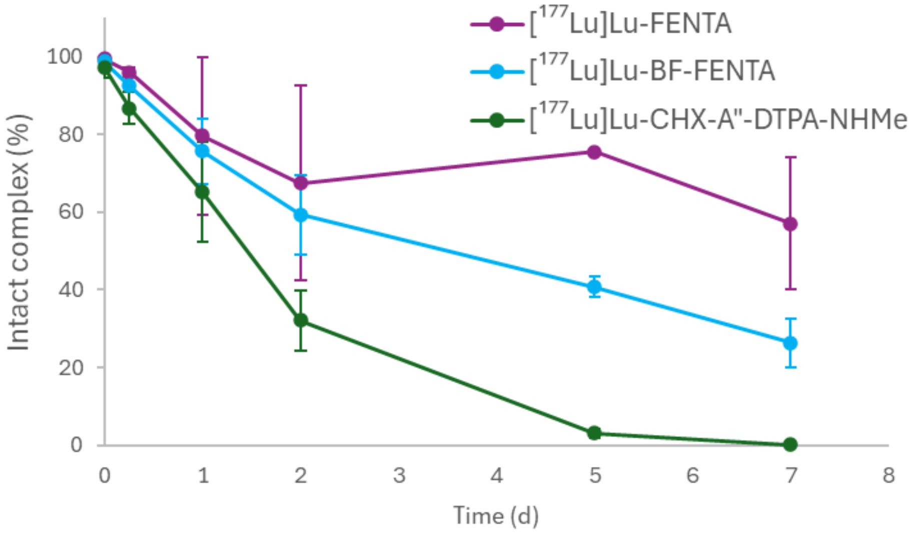

Fig. S11 DTPA challenge of [^{177}Lu]Lu^{3+} complexes of H_4FENTA, BF-FENTA and CHX-A"-DTPA in the presence of a 1000-fold DTPA (DTPA to ligand ratio). All reactions were carried out in acetate buffer (0.25 M, pH 4.7, 75 µL) with 75 MBq (15 µL) at an AMA of 50 MBq/nmol in the presence of AA (33 mM), at 40°C for 15 min. After radiolabeling, the solution was diluted 6-fold with acetate buffer. The percentage of intact complex was monitored by radio-HPLC analysis (n = 2).

Bismuth-213

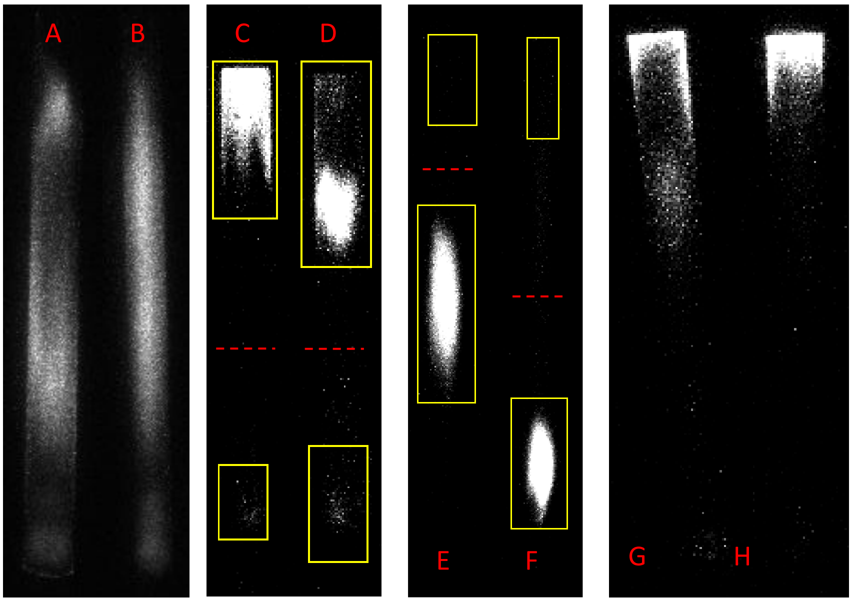

Fig. S12 iTLC strips of [^{213}Bi]Bi-FENTA (A), [^{213}Bi]Bi-BF-FENTA (B), [^{213}Bi]Bi-CHX-A''-DTPA-NHMe (C), and [^{213}Bi]Bi-DOTA (D), developed with ACN:H_2O (3:1) as the mobile phase, and iTLC strips of [^{213}Bi]Bi-FENTA (E), [^{213}Bi]Bi-BF-FENTA (F), [^{213}Bi]Bi-CHX-A''-DTPA-NHMe (G), and [^{213}Bi]Bi-DOTA (H), developed with citrate buffer (0.1 M, pH 4.8) as the mobile phase. iTLCs strips were visualized by autoradiography. TLC plates were cut along the red dotted line prior to activity quantification using a gamma counter.

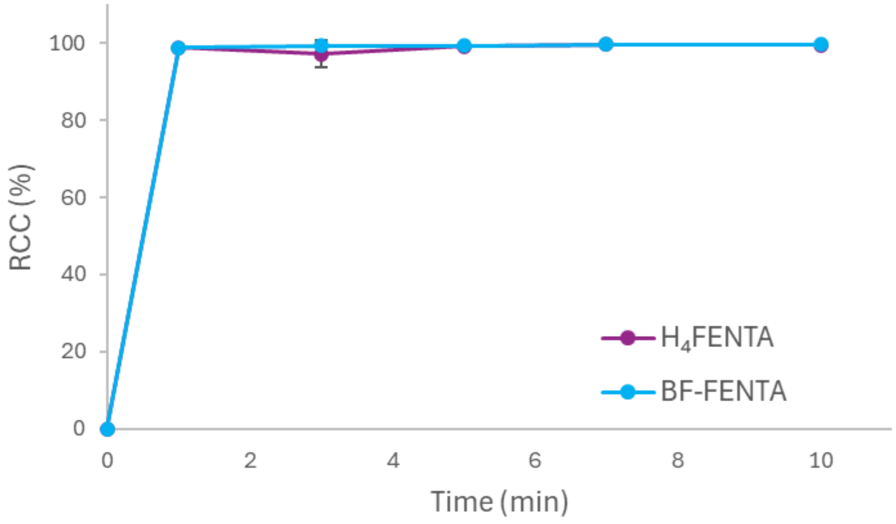

Fig. S13 Association experiment of H_4FENTA and BF-FENTA with [^{213}Bi]Bi^{3+} (1.3 – 1.4 MBq, 40 µL) in 1 M NaOAc buffer (pH 4.8, 20 µL) in the presence of AA (30 mM), performed at 40 °C for 5 min. The percentage of radiometal bound was quantified using a gamma counter (cut and count method; n = 3).

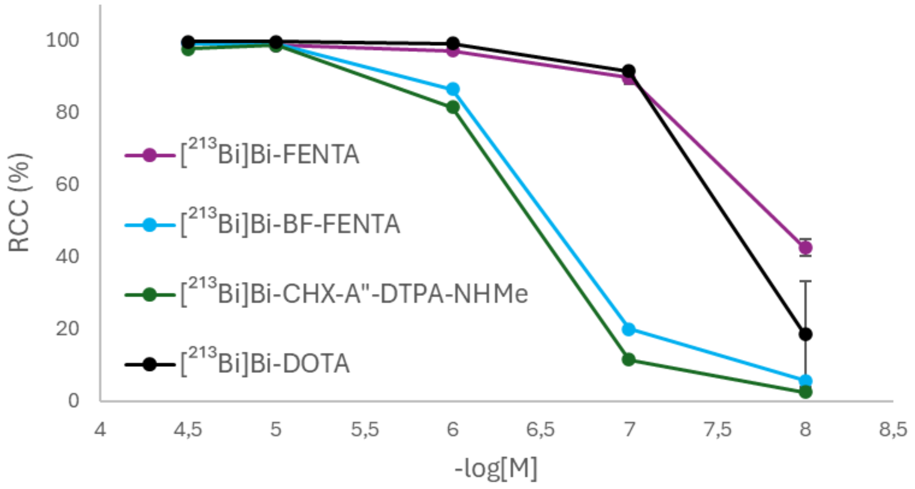

Fig. S14 Data of the molar activity escalation experiment (**Fig. 4**) expressed as function of the molar concentration of H_4FENTA, BF-FENTA, DOTA, and CHX-A"-DTPA-NHMe. All reactions were carried out with [^{213}Bi]Bi^{3+} (1.1 – 1.2 MBq, 40 µL) in 1 M NaOAc buffer (pH 4.8, 20 µL) in the presence of AA (30 mM), at 40 °C for 5 min. For DOTA, labeling was performed at 90 °C for 30 min. The percentage of radiometal bound was quantified using a gamma counter (cut and count method; n = 3).

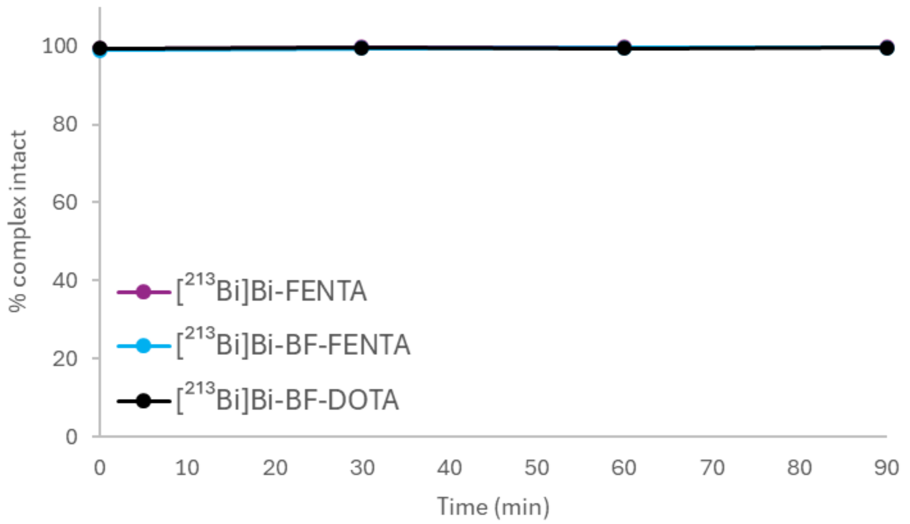

Fig. S15: Stability of [^{213}Bi]Bi^{3+}-complexes in NaOAc buffer (1 M, pH 4.8) at RT. All reactions were carried out with [^{213}Bi]Bi^{3+} (1.2 – 1.4 MBq, 40 µL) in 1 M NaOAc buffer (pH 4.8, 20 µL) in the absence of AA, at 40 °C for 5 min. For DOTA, labeling was performed at 90 °C for 30 min. The percentage of radiometal bound was quantified using a gamma counter (cut and count method; n = 3).

NMR data

*Tetra-tert-butyl 2,2',2'',2'''-(((1,10-phenanthroline-2,9-diyl)bis(methylene))bis(azanetriyl))tetra-acetate (**3**)*

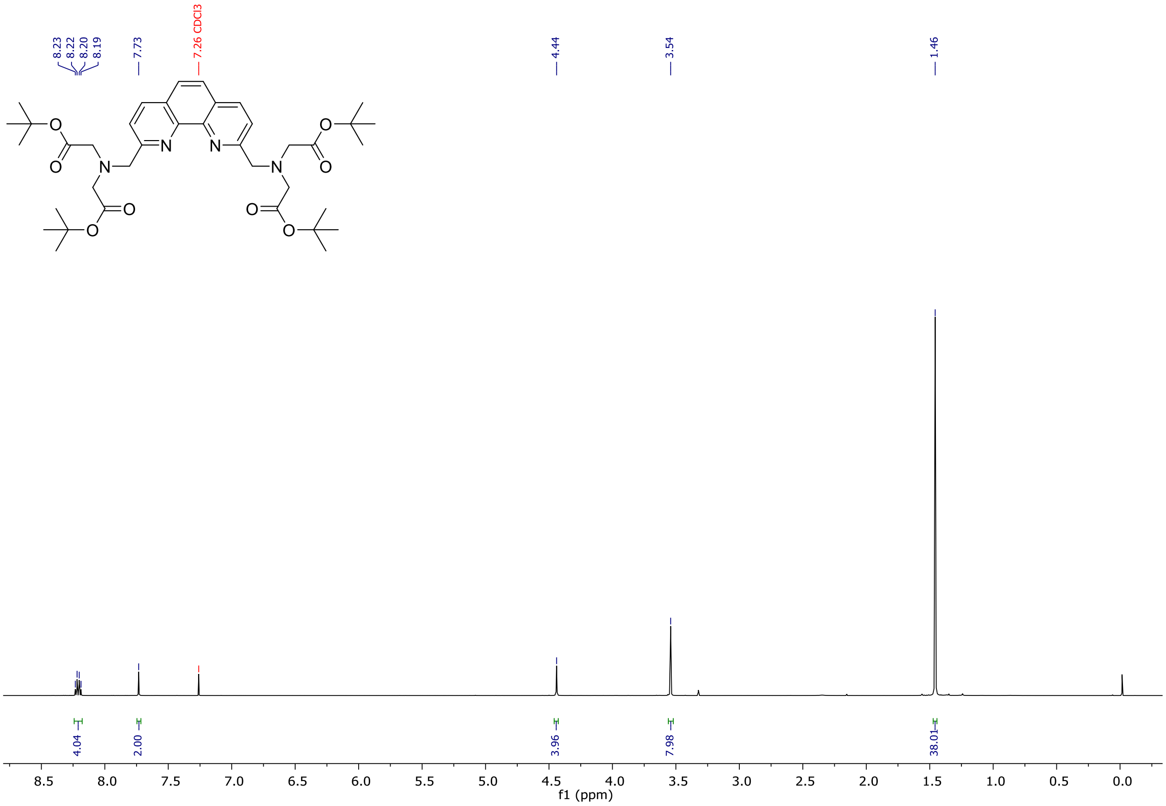

Fig. S16: ^{1}H NMR spectrum (600 MHz, $CDCl_3$) of **3**.

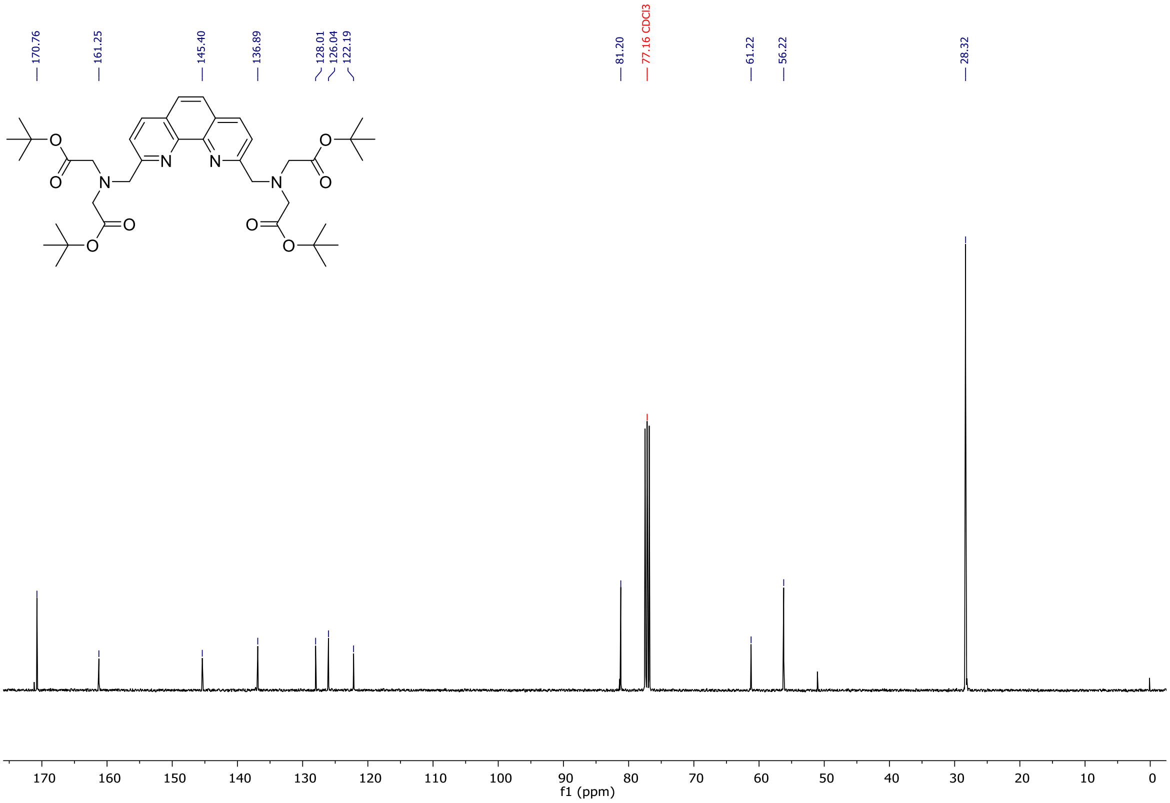

Fig. S17: ^{13}C NMR spectrum (101 MHz, $CDCl_3$) of **3.**

2,2',2'',2'''-(((1,10-Phenanthroline-2,9-diyl)bis(methylene))bis(azanetriyl))tetraacetic acid (**H_4FENTA**)

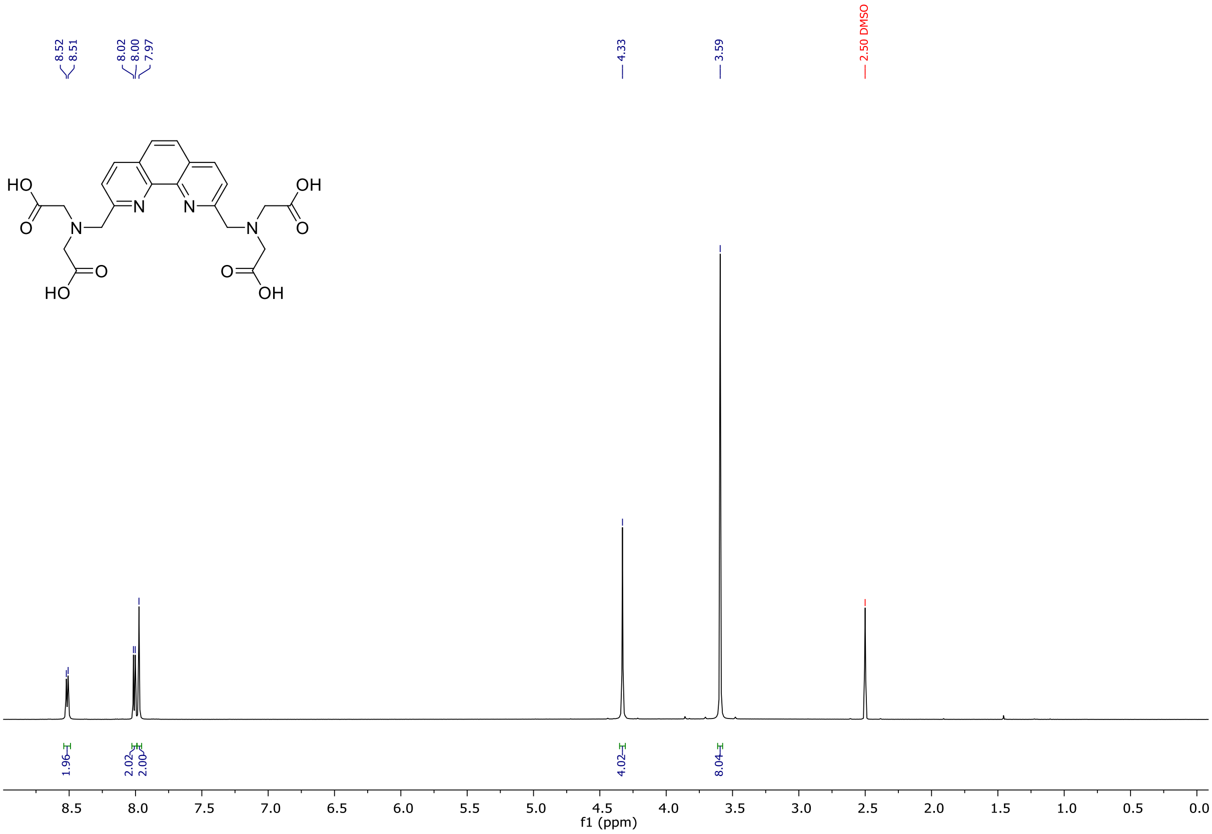

Fig. S18: ^{1}H NMR spectrum (600 MHz, DMSO-d_6) of **H_4FENTA**.

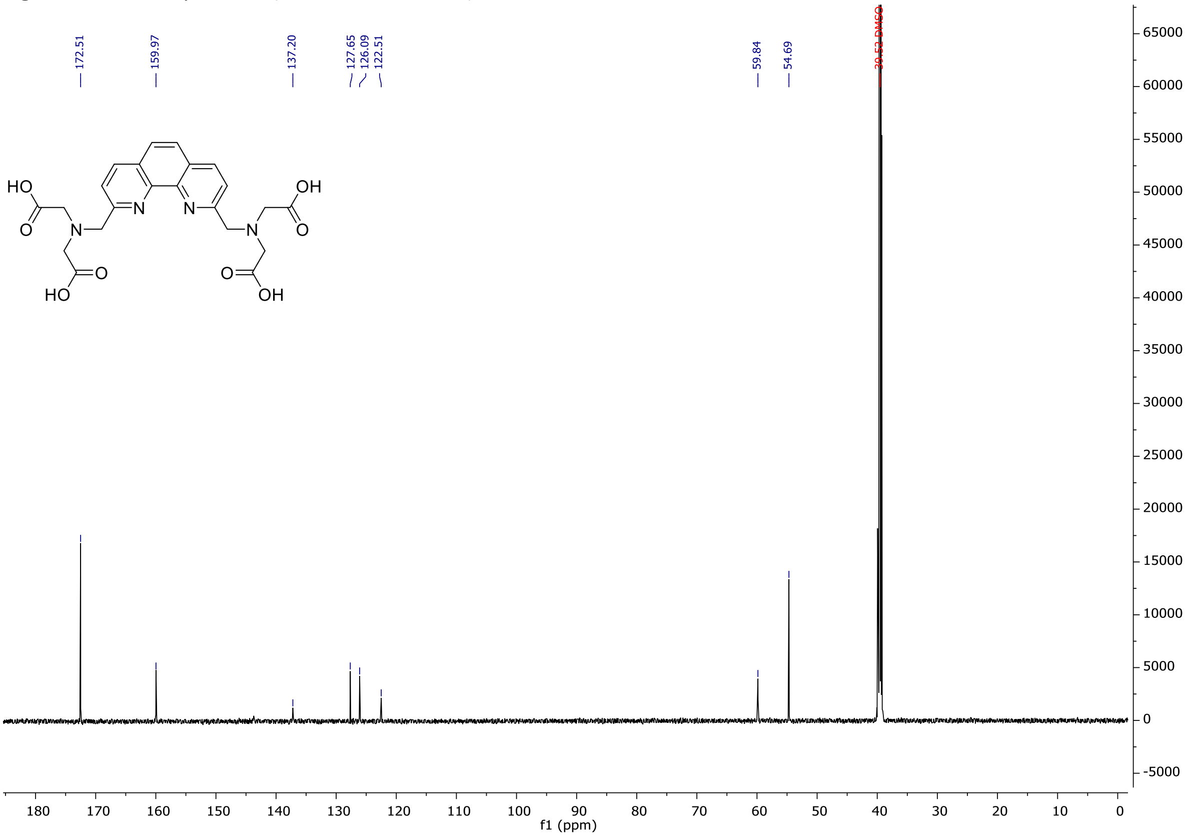

Fig. S19: ^{13}C NMR spectrum (151 MHz, DMSO-d_6) of **H_4FENTA**.

*N-(2-(2-Azidoethoxy)ethyl)-2-((2-nitrophenyl)sulfonamido)acetamide (**6**)*

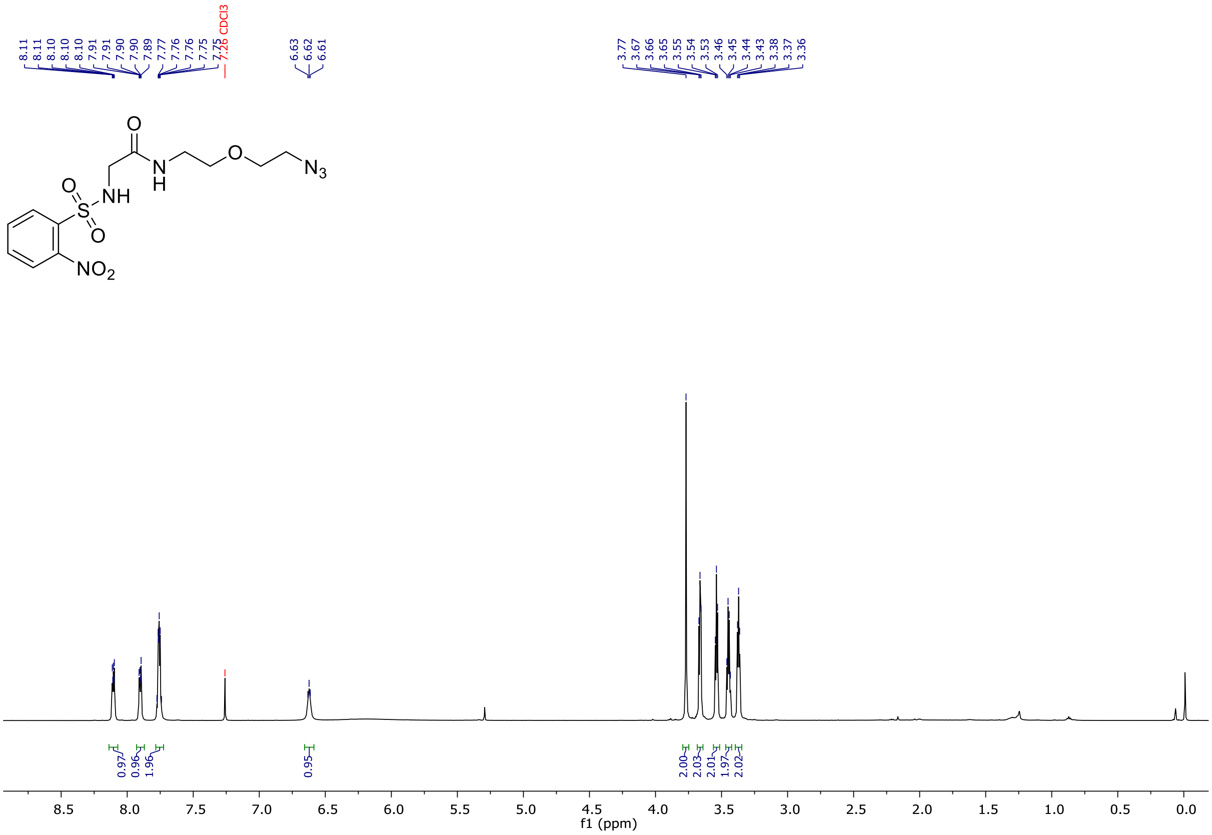

Fig. S20: ^{1}H NMR spectrum (600 MHz, $CDCl_3$) of **6.**

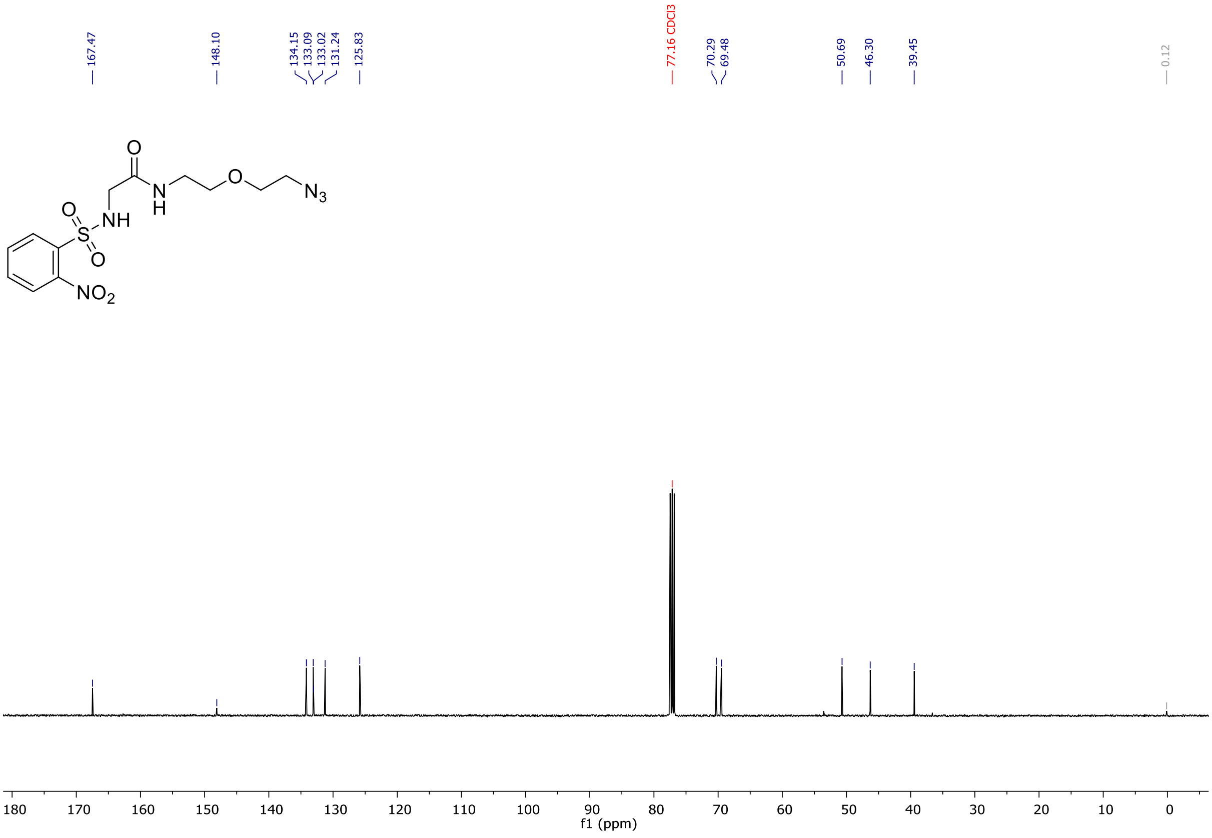

Fig. S21: ^{13}C NMR spectrum (101 MHz, $CDCl_3$) of **6.**

Tert-butyl N-(2-((2-(2-azidoethoxy)ethyl)amino)-2-oxoethyl)-N-((2-nitrophenyl)sulfonyl)glycinate ***(8)***

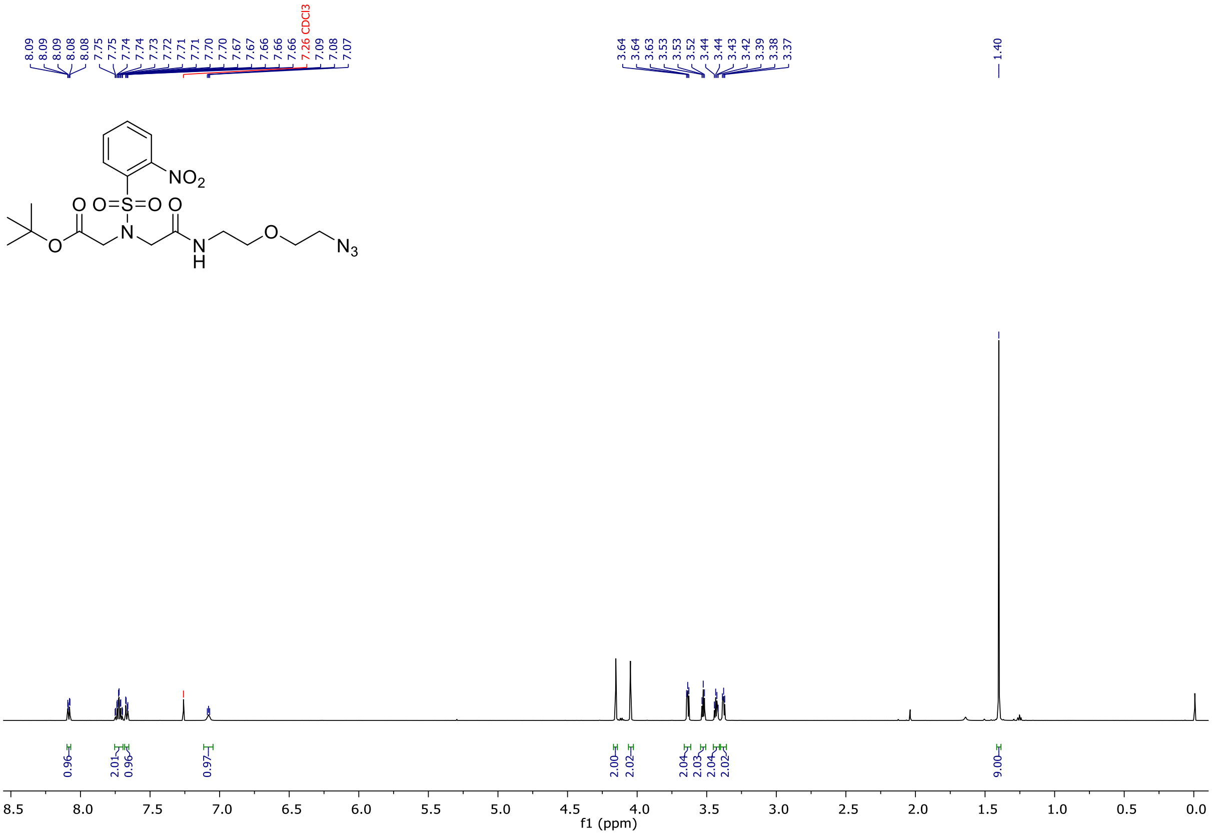

Fig. S22: ^{1}H NMR spectrum (600 MHz, $CDCl_3$) of **8.**

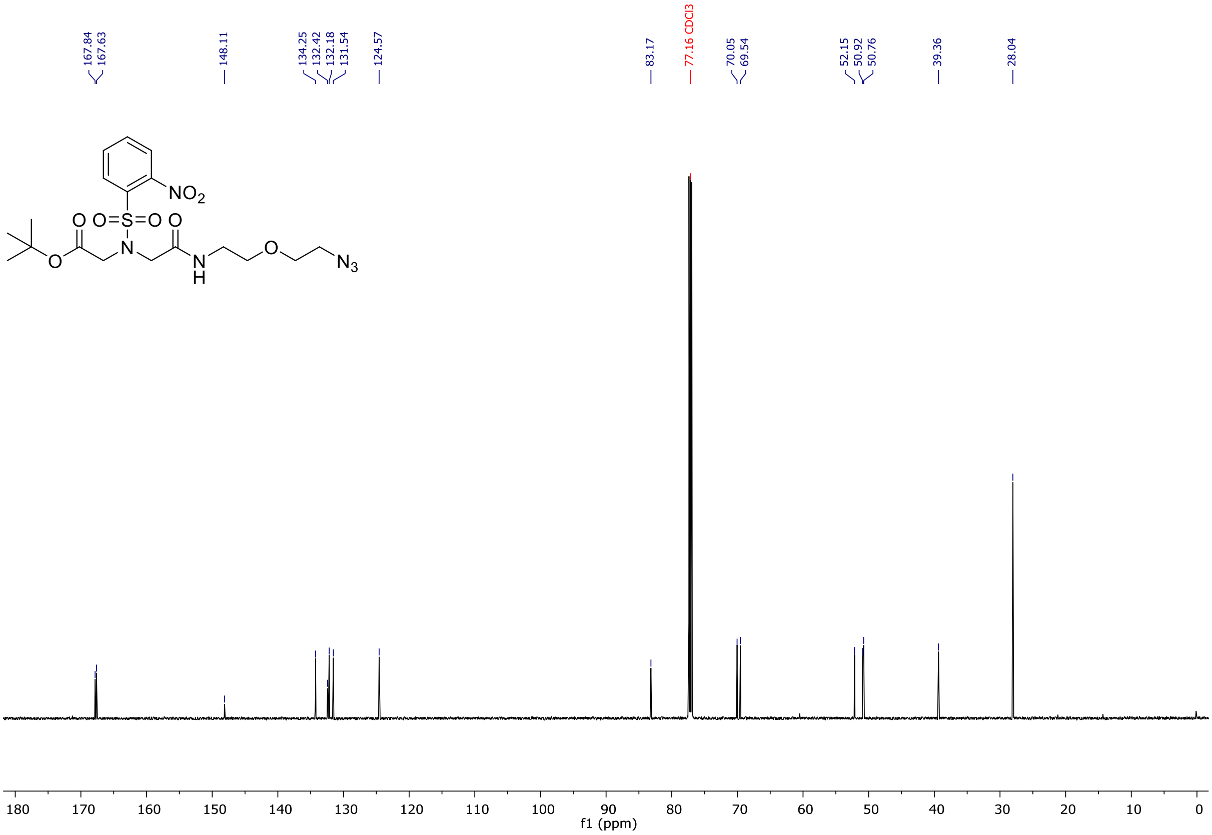

Fig. S23: ^{13}C NMR spectrum (151 MHz, $CDCl_3$) of **8.**

*Tert-butyl (2-((2-(2-azidoethoxy)ethyl)amino)-2-oxoethyl)glycinate (**9**)*

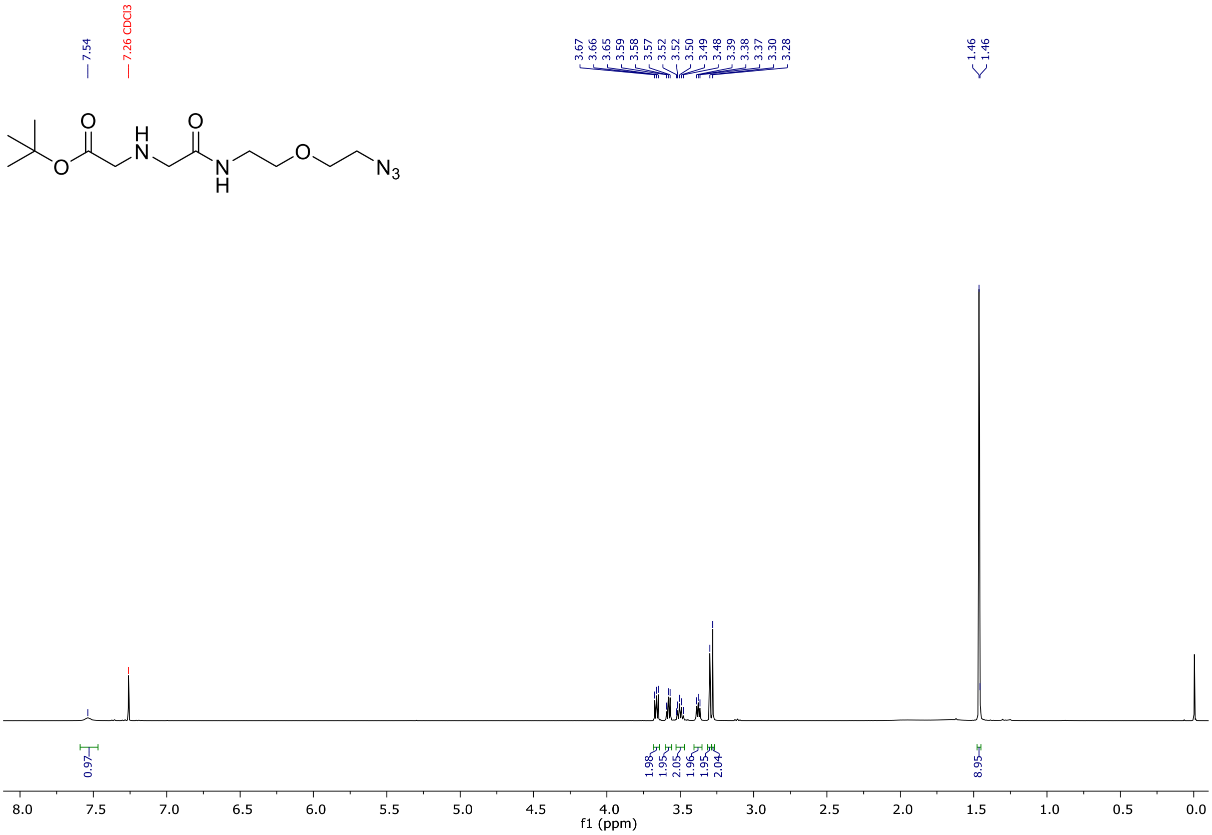

Fig. S24: ^{1}H NMR spectrum (400 MHz, $CDCl_3$) of **9.**

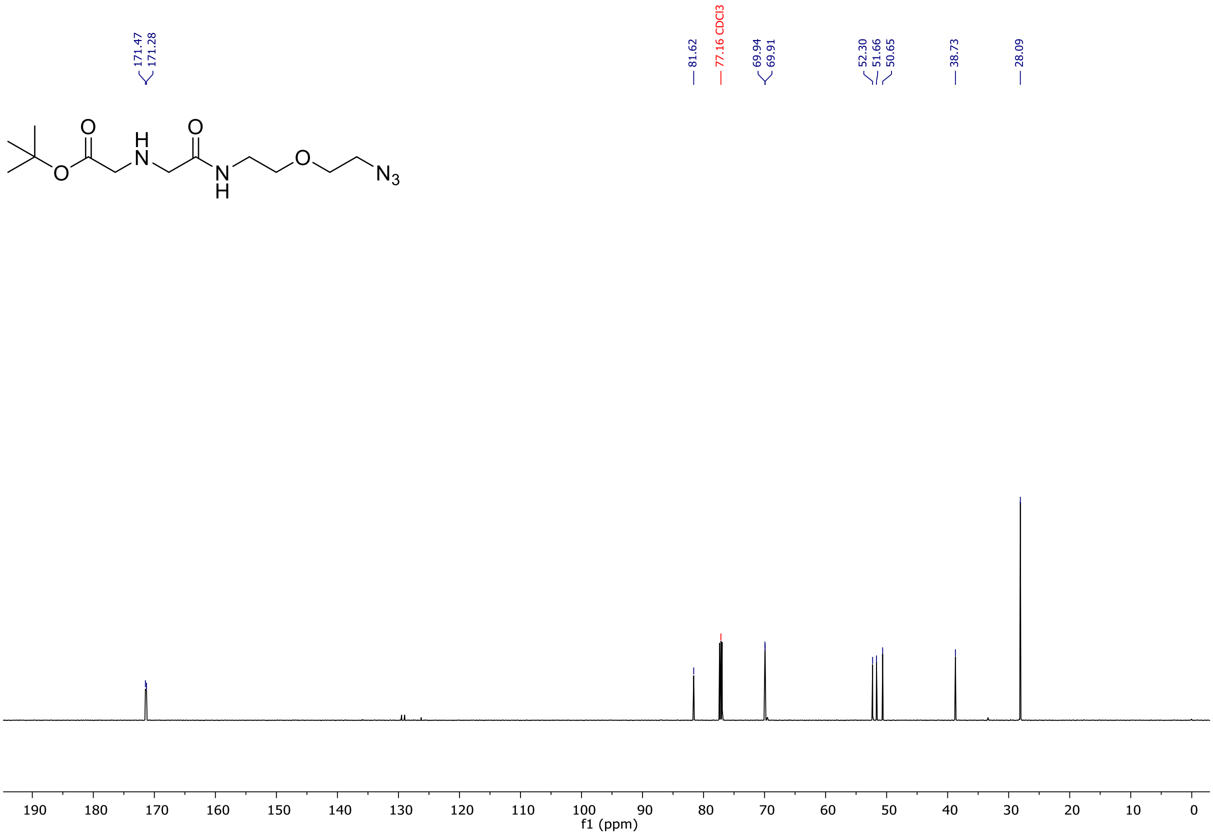

Fig. S25: ^{13}C NMR spectrum (151 MHz, $CDCl_3$) of **9**.

*Di-tert-butyl 2,2'-(((9-(chloromethyl)-1,10-phenanthrolin-2-yl)methyl)azanediyl)diacetate (**10**)*

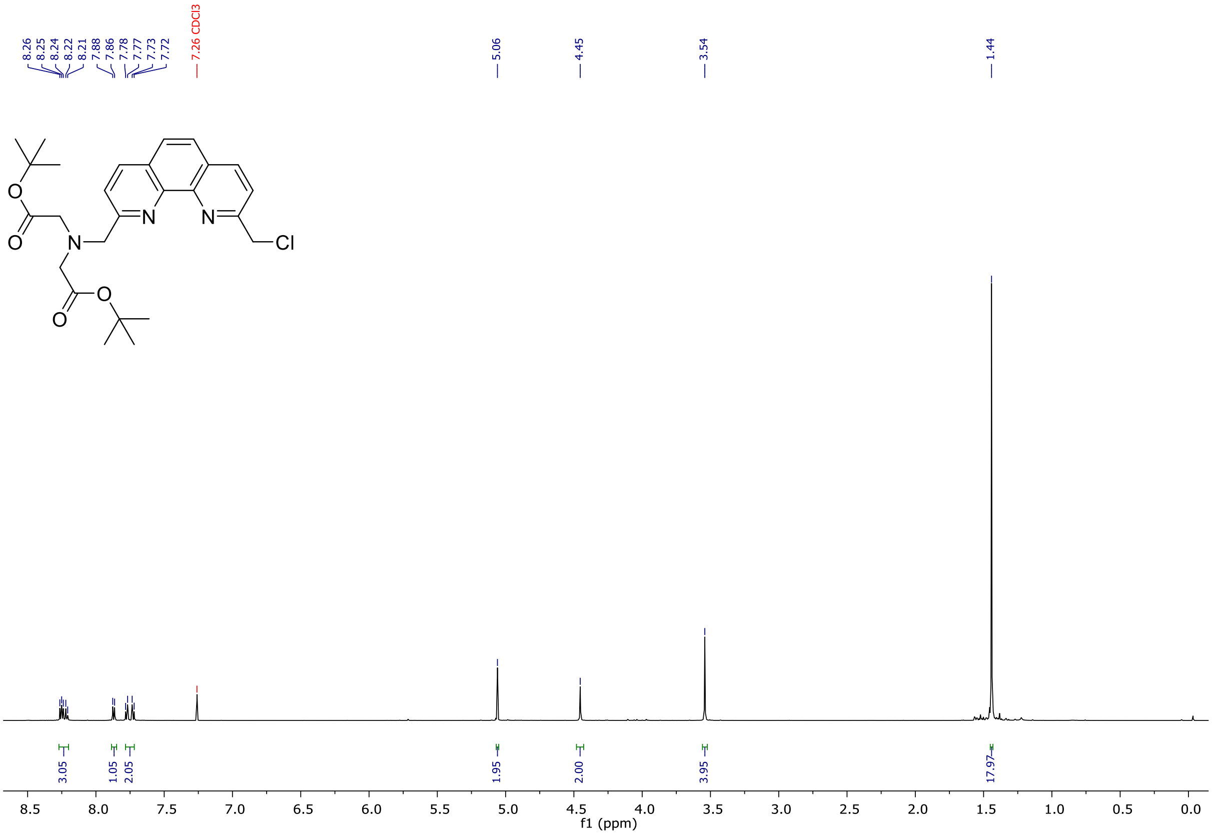

Fig. S26: ^{1}H NMR spectrum (600 MHz, $CDCl_3$) of **10.**

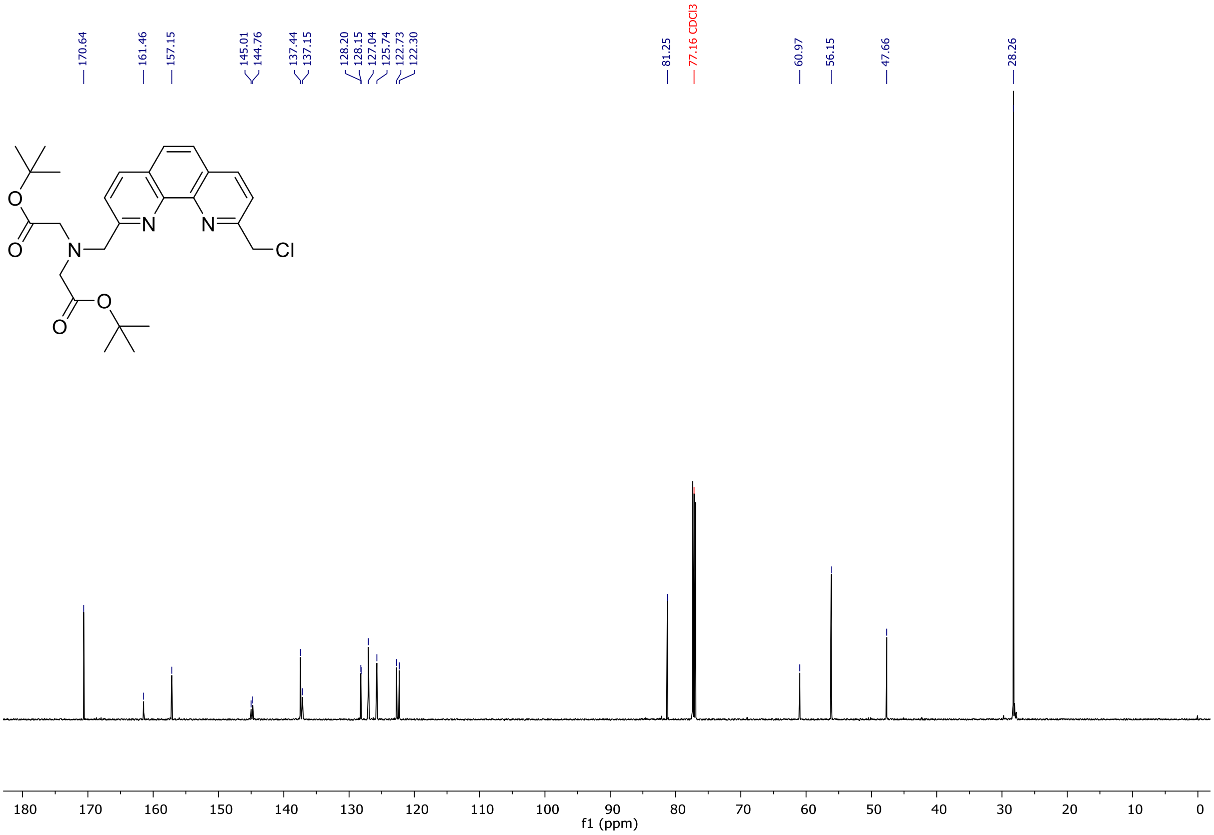

Fig. S27: ^{13}C NMR spectrum (151 MHz, $CDCl_3$) of **10.**

*Di-tert-butyl 2,2'-(((9-(((2-((2-(2-azidoethoxy)ethyl)amino)-2-oxoethyl)(2-(tert-butoxy)-2-oxoethyl)amino)methyl)-1,10-phenanthrolin-2-yl)methyl)azanediyl)diacetate (**11**)*

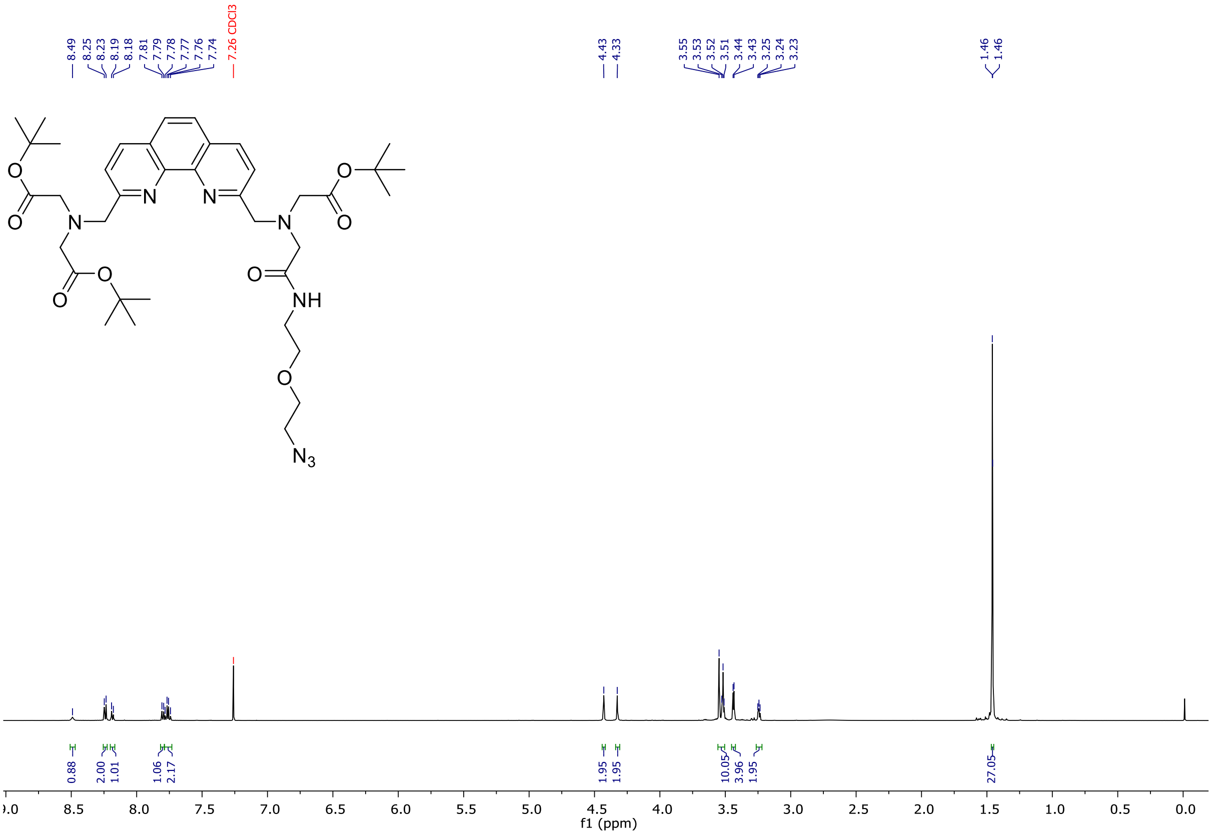

Fig. S28: ^{1}H NMR spectrum (600 MHz, $CDCl_3$) of **11.**

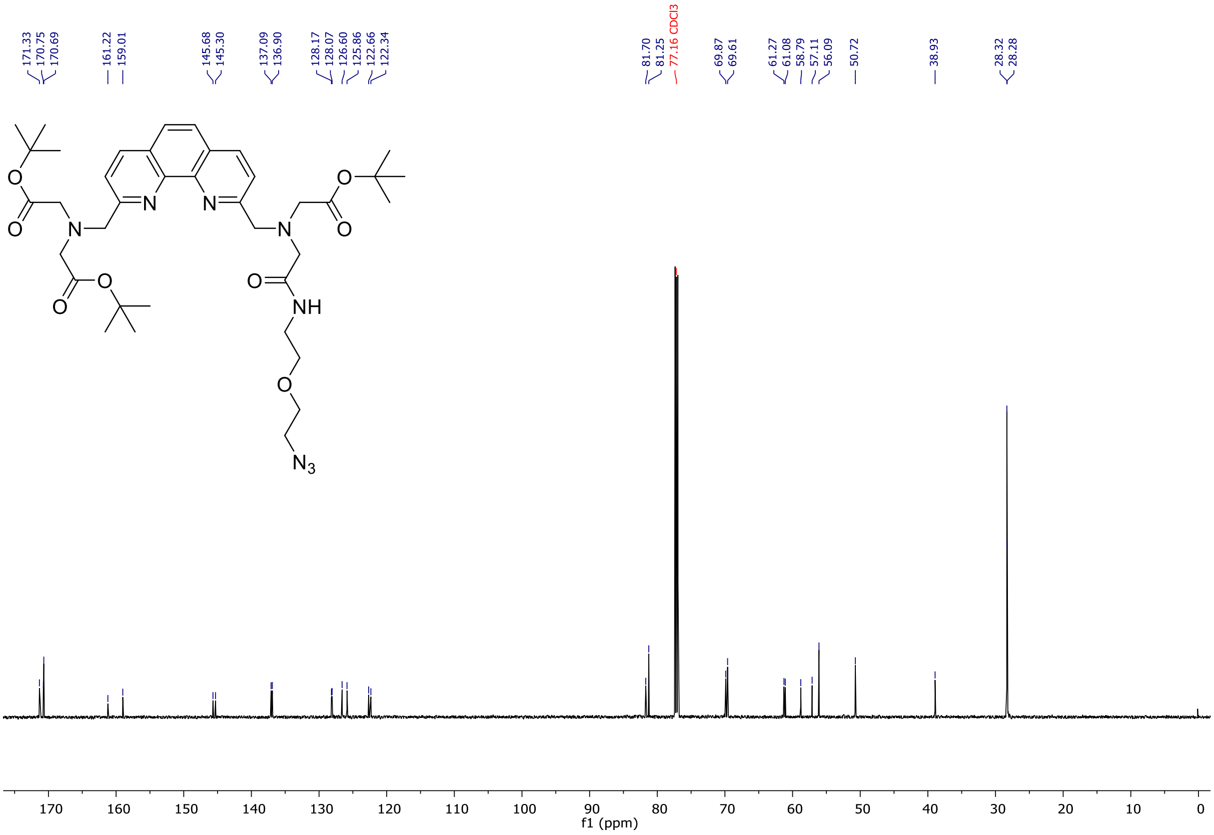

Fig. S29: ^{13}C NMR spectrum (151 MHz, $CDCl_3$) of **11.**

*2,2'-(((9-(((2-((2-(2-Azidoethoxy)ethyl)amino)-2-oxoethyl)(carboxymethyl)amino)methyl)-1,10-phenanthrolin-2-yl)methyl)azanediyl)diacetic acid (**BF-FENTA**)*

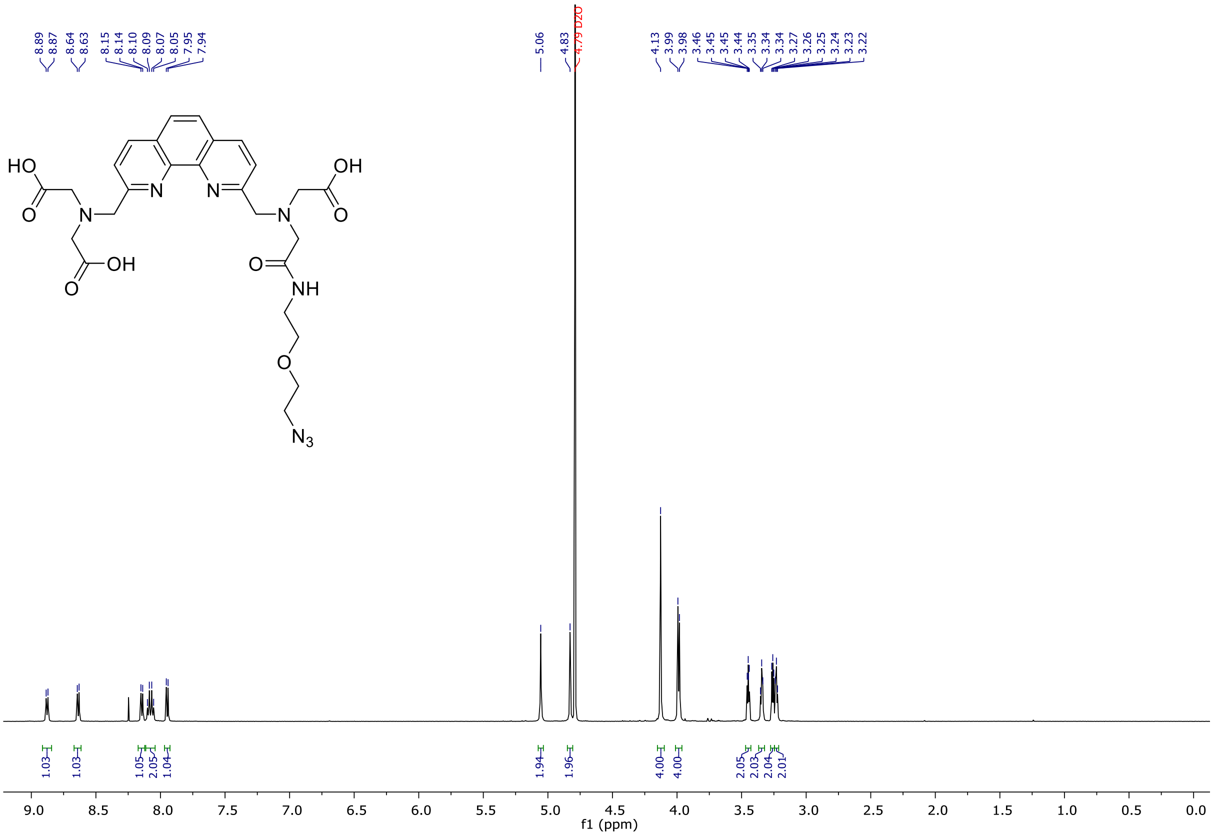

Fig. S30: ^{1}H NMR spectrum (600 MHz, D_2O) of **BF-FENTA.**

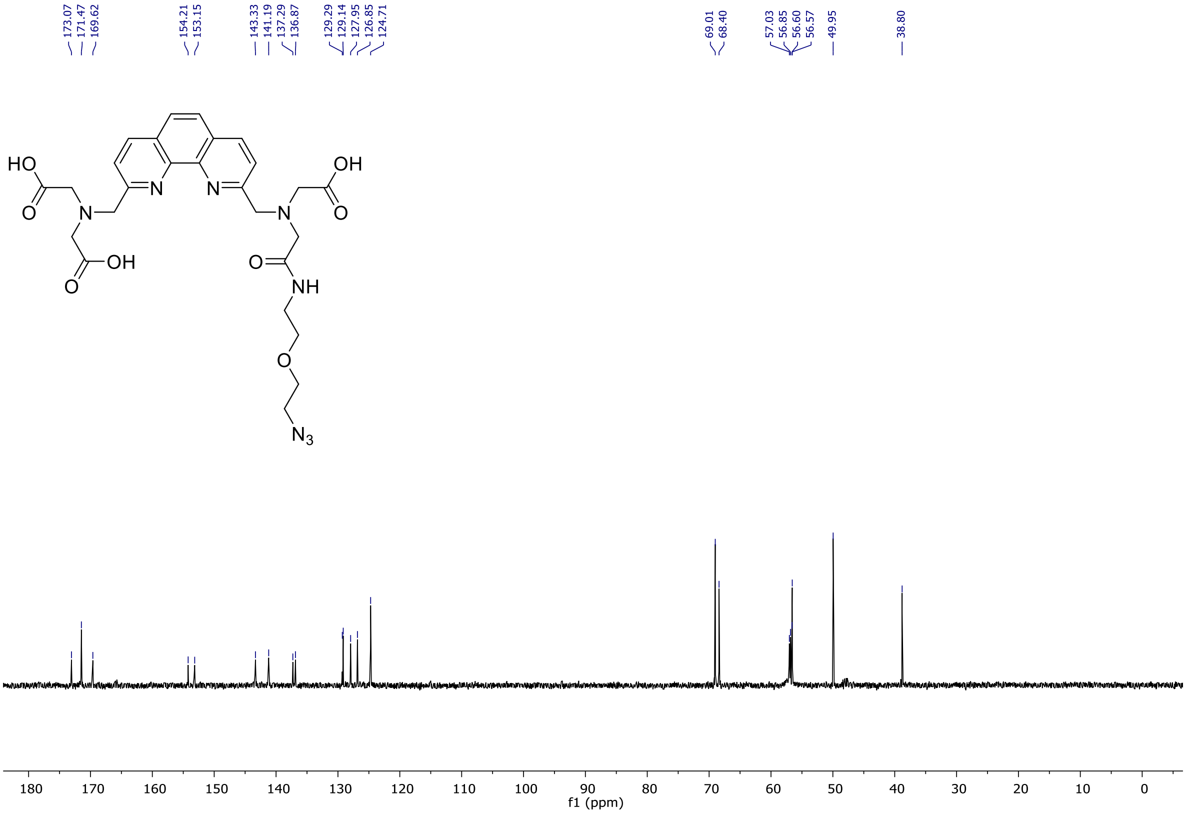

Fig. S31: ^{13}C NMR spectrum (101 MHz, D_2O) of **BF-FENTA.**
